# Multimodal Profiling Reveals Distinct Endothelial Activation Pathways Regulated by Flow and Heparan Sulfate

**DOI:** 10.1007/s12195-026-00884-3

**Published:** 2026-02-01

**Authors:** Ian C. Harding, Nicholas R. O’Hare, Ira M. Herman, Eno E. Ebong

**Affiliations:** 1 Department of Bioengineering, Northeastern University, Boston, MA USA; 2 Department of Chemical Engineering, Northeastern University, 360 Huntington Avenue, 129 Interdisciplinary Science and Engineering Complex, Boston, MA 02115 USA; 3 Department of Genetics, Molecular and Developmental Biology, Tufts University School of Medicine, Boston, MA USA; 4 Center for Innovations in Wound Healing Research, Tufts University School of Medicine, Boston, MA USA; 5 Tissue Health Plus, Inc., Fort Worth, TX USA; 6 Department of Neuroscience, Albert Einstein College of Medicine, New York, NY USA

**Keywords:** Endothelial glycocalyx, Reactive oxygen species, Endothelial inflammation, Mechanotransduction, RNA sequencing

## Abstract

**Purpose:**

Atherosclerotic cardiovascular disease originates from endothelial dysfunction, characterized by a shift toward a pro-inflammatory state and increased production of reactive oxygen species (ROS). This dysfunction occurs under adverse mechanical conditions, such as blood flow oscillation, multi-directionality, recirculation, shear stress gradients, and low or stagnation flows. This study investigates how degradation of heparan sulfate (HS), a major component of the endothelial glycocalyx, drives the transition of endothelial cells from a functional, anti-inflammatory, and antioxidant phenotype under streamlined flow conditions to a dysfunctional, pro-inflammatory, and pro-oxidant phenotype when flow is stagnant. Pro-inflammatory and pro-oxidant endothelial behavior precedes atherosclerosis development.

**Methods:**

Human aortic endothelial cells were exposed to uniform shear stress (14 dynes/cm^2^) to model healthy endothelium. Unhealthy conditions were simulated via static conditions (0 dynes/cm^2^) or enzymatic HS degradation using heparinase III. Endothelial cell phenotype was assessed using fluorescent labeling, confocal microscopy, Western blotting, and RNA sequencing.

**Results:**

Endothelial cells conditioned by 14 dynes/cm^2^ shear stress without heparinase III exhibited low expression of pro-inflammatory genes (HIF1A, VCAM1, and IL1B), minimal ROS production, and up-regulation of Kruppel-like transcription factors. Under the same flow conditions, HS degradation via heparinase III induced an inflammatory phenotype, resembling responses observed at 0 dynes/cm^2^ shear stress, while ROS levels remained largely unaffected.

**Conclusions:**

The endothelial glycocalyx is a protective, dynamic, and complex structure, with HS as a key component. This study demonstrates that intact HS mitigates endothelial dysfunction by suppressing inflammation linked to flow-dependent atherosclerosis, but not ROS production. Future research will focus on translating these findings into HS-targeted therapies for atherosclerotic cardiovascular disease.

**Supplementary Information:**

The online version contains supplementary material available at 10.1007/s12195-026-00884-3.

## Introduction

**Fig. 1:**
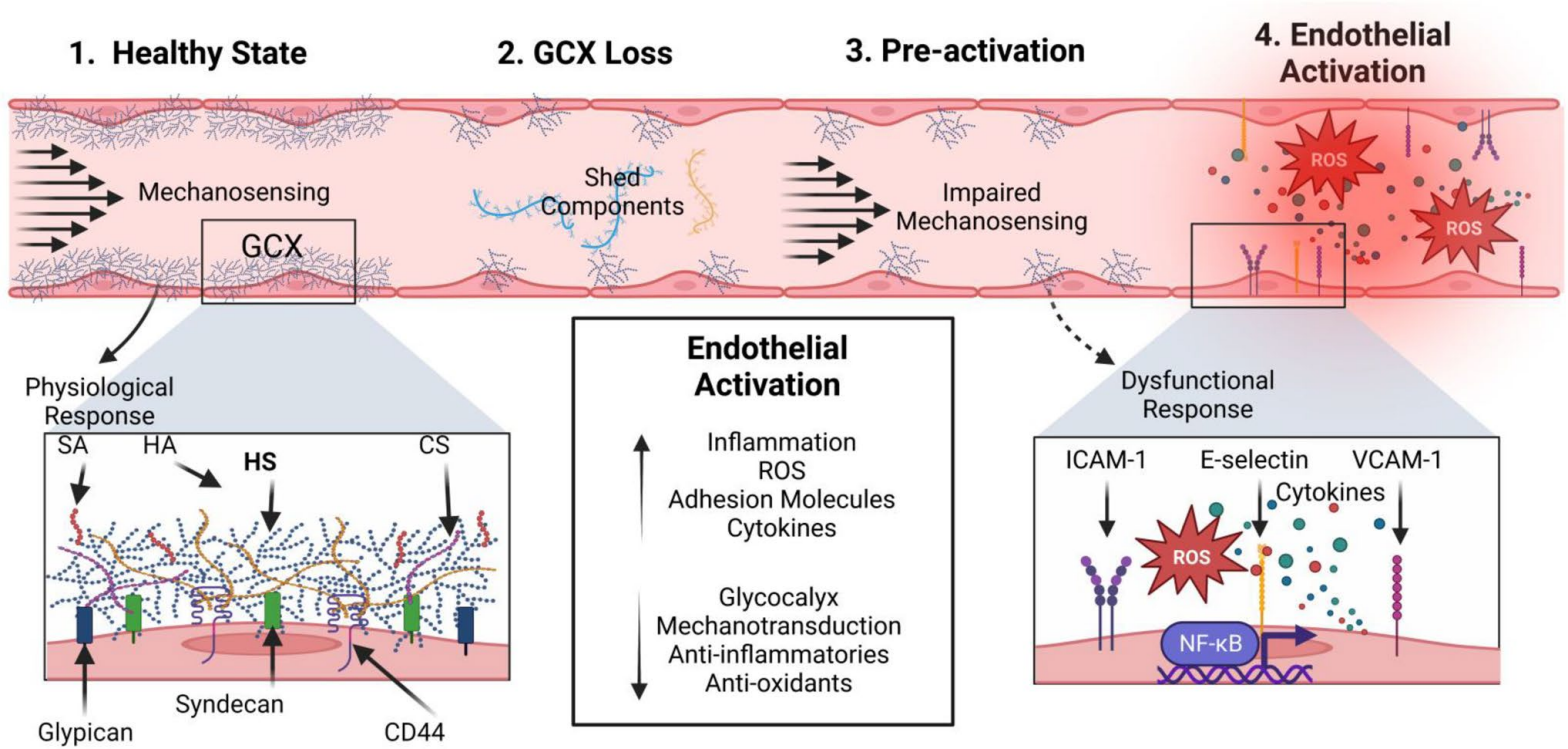
*Graphical abstract of endothelial activation (this figure was created using BioRender.com). (1.) Healthy State*: In physiological conditions, fluid shear stress on the endothelial glycocalyx (GCX) transduces a cellular response to balance the cell’s redox and inflammatory state, deterring endothelial activation. A simplified GCX is depicted in the box containing key components heparan sulfate (HS), hyaluronic acid (HA), chondroitin sulfate (CS), sialic acid (SA), syndecan, glypican, and CD44. *(2.) GCX Loss:* In diseased states, components like HS are cleaved from the GCX and circulate freely in the blood stream. *(3.) Pre-activation:* Due to the shed GCX, cells are not given the proper mechanical signals to regulate endothelial activation. *(4.) Endothelial Activation:* In this diseased endothelial phenotype, inflammation and ROS are increased, enabling activation of Nf-κb-based cytokine generation, increased adhesion molecule expression, and a dysfunctional endothelium

Endothelial cell activation is most broadly characterized by a transition from an anti- to a pro-inflammatory state and is associated with a number of cardiovascular diseases including atherosclerosis [1, 2], kidney disease [3, 4], and cerebral small vessel disease [5, 6]. On the molecular level, endothelial activation is denoted by the increased expression of pro-inflammatory molecules such as cellular adhesion molecules [7, 8], pro-inflammatory transcription factors [9, 10], and cytokines (Fig. 1) [11]. To illustrate, activated endothelial cells are characterized by increased expression of the pro-inflammatory transcription factor Nf-κb [10], increased expression of the adhesion molecules VCAM-1, ICAM-1, and E-selectin [7, 12], and increased production of cytokines such as interleukin-1β [13]. Endothelial activation is also associated with the production of reactive oxygen species (ROS), such as superoxide [14] and hydrogen peroxide [15]. The regulation of these ROS involves the balanced expression and function of oxidant proteins, such as the nicotinamide adenine dinucleotide phosphate oxidase (Nox) family of proteins, and antioxidant proteins, such as glutathione reductase and heme oxygenase [16]. Expression of these proteins, and in turn the regulation of cells’ redox states, is mediated upstream by the ROS-reducing transcription factor nuclear factor erythroid 2-related factor 2 (Nrf2) [17, 18]. Activation of Nrf2 promotes a shift towards antioxidant activity characterized by the decreased expression of oxidant proteins such as Nox2 [19].

Endothelial phenotype is partially regulated by blood flow conditions, where fluid shear stress governs endothelial function through mechanotransduction, the conversion of mechanical forces into biochemical signals. Specifically, uniform physiological shear stress exposure has been shown to promote endothelial cell expression of anti-inflammatory genes, such as endothelial nitric oxide synthase, while reducing the expression of pro-inflammatory genes, such as the adhesion receptor ICAM-1 [20, 21]. Similarly, the production of ROS has also been shown to be regulated by the application of uniform physiological shear stress. The temporal dynamics of flow-induced ROS regulation are generally accepted to be biphasic: short-term flow exposure (minutes to 6 hours) can transiently increase ROS production [22–26] while prolonged exposure (>16 hours) consistently reduces ROS levels and enhances antioxidant capacity [27, 28]. This biphasic response reflects a transition from initial oxidative signaling to sustained antioxidant adaptation, highlighting the importance of flow exposure duration in determining endothelial redox state.

The endothelial glycocalyx (GCX), a glycoprotein-proteoglycan layer extending from the endothelial membrane, is a known mechanotransducer that promotes proper function of endothelial cells (Fig. 1) [29]. Degradation of the GCX has been shown to impair mechanotransduction, marked by decreased nitric oxide (NO) production [30, 31], increased expression of cellular adhesion molecules [32, 33], and increased activation of pro-inflammatory transcription factors [32]. Major GCX components implicated in mechanotransduction include its core proteins, mainly syndecan and glypican, the protein receptor CD44, and the attached glycosaminoglycans heparan sulfate (HS), hyaluronic acid, and chondroitin sulfate. The GCX is also modified with sialic acid sugar residues, which impart a net negative charge to the structure [29]. HS is the most abundant glycosaminoglycan of the GCX and was therefore the GCX component of interest in this exploratory study.

GCX degradation is associated with classical markers of pro-inflammatory endothelial activation, such as increased adhesion molecule expression [30–33]. Also, it was previously reported that degradation of the HS GCX component leads to increased ROS production [34], although these findings have not been further validated. High-throughput analyses of how HS degradation affects endothelial inflammatory and oxidative states remain unexplored. Such investigations would provide a deeper understanding of GCX’s role in endothelial function regulation, and its molecular mechanisms of action. Furthermore, since HS is involved in numerous force-independent signaling pathways [35–39], it is important to determine whether its regulation of inflammatory and oxidative endothelial activation occurs through force-dependent or force-independent mechanisms. This motivates the present study, aimed at investigating the impact of shear stress application and HS degradation on endothelial activation through quantifying the expression of endothelial inflammatory markers and ROS production.

We hypothesized that uniform physiological flow exposure would promote an anti-inflammatory, anti-oxidant endothelial state, while degradation of the GCX, specifically of its HS component, would lead to increased production of ROS and increased expression of pro-inflammatory genes. To test this hypothesis, we employed a comprehensive approach combining parallel plate flow endothelial cell culture modeling, RNA-sequencing (RNA-seq), fluorescence microscopy, and Western blotting. The insights from this study, outlined in the following sections, could contribute to the development of targeted therapies to mitigate endothelial activation and ultimately prevent downstream cardiovascular diseases.

## Materials and Methods

### Cell Culture

Primary human aortic endothelial cells (HAECs) were purchased from PromoCell (Heidelberg, Germany) and used between passages 4 and 8. Cells were cultured in PromoCell Endothelial Cell Growth Media MV2 supplemented with penicillin-streptomycin (100 U/mL and 100 μg/mL, respectively) and the MV2 SupplementPack, which included fetal calf serum (0.05 mL/mL), recombinant human epithelial growth factor (5 ng/mL), recombinant human basic fibroblast growth factor (10 ng/mL), insulin-like growth factor (20 ng/mL), recombinant human vascular endothelial growth factor 165 (0.5 ng/mL), ascorbic acid (1 μg/mL), and hydrocortisone (0.2 μg/mL). Cells were grown in a humidified incubator at 37 °C and 5% CO_2_. For shear stress and static experiments (described below), HAECs were plated on fibronectin-coated (2 μg/cm^2^) glass coverslips at a concentration of 2.5 × 10^5^ cells/mL and cultured for 3-4 days before the onset of experiments. For all experiments, media was supplemented with 0.5% bovine serum albumin to promote GCX stabilization.

### Shear Stress Application

Confluent HAEC monolayers were exposed to shear stress in a parallel-plate flow chamber that was adapted from previous work [31] to generate uniform flow. The parallel-plate flow chamber was designed using SolidWorks, and shear stress validation was performed using SolidWorks Flow Simulation. Channel dimensions were 90 mm in length, 13.5 mm in width, and 1 mm in height. The flow loop contained the parallel-plate flow chamber itself, tubing, a medium reservoir to allow for CO_2_ diffusion, and a pulse dampener to remove pulsatility generated by the peristaltic pump. The total system volume was 60 mL based on the chamber, reservoir, dampener, and tubing. Cells were exposed to flow for 24 hours at an eventual 14 dynes/cm^2^, administered using a peristaltic pump set to apply a flow rate of 120 mL/min. To allow for cell adaptation to the flow environment, cells were initially exposed to a shear stress of 4.66 dynes/cm^2^ for one hour, followed by 9.33 dynes/cm^2^ for one hour, and finally 14 dynes/cm^2^ for the remaining 22 hours. The applied flow was steady and unidirectional, generating a laminar profile (Re < 1) characteristic of atheroprotective shear conditions. During experimentation, the entire flow setup, except for the peristaltic pump, was contained within a humidified incubator at 37 °C and 5% CO_2_. For reference and to establish a baseline in the absence of flow-mediated mechanotransduction, there were also confluent HAEC monolayers exposed to static conditions (0 dynes/cm^2^). The static samples were cultured separately in standard ventilated cell culture dishes, maintained under identical incubator conditions as the flow experiments. Although not connected to the flow loop, the incubator provided the static samples with controlled humidity, 37 °C temperature, and 5% CO_2_ to ensure proper oxygenation and pH balance. For both flow‑conditioned and static samples, fresh medium of sufficient volume was provided at the start of the 24‑hour experiment to ensure adequate nutrients and limit metabolite buildup, thereby reducing potential confounding effects on gene expression independent of shear.

### Enzymatic Glycocalyx (GCX) Degradation

The HS component of the GCX was enzymatically degraded using heparinase III (Hep III). Cells were incubated in cell medium in the presence of 250 μIU(micro‑International Unit)/mL Hep III (Ibex; Montreal, Canada) for 24 hours under either the static or shear stress experimental conditions. The Hep III dosage was determined via a dose-response test in which HS degradation was confirmed using immunofluorescence cytochemistry, fluorescence microscopy, and fluorescence image analysis (Supplemental Fig. 1). A concentration range of 62.5 μIU/mL to 500 μIU/mL was tested and 250 μIU/mL produced ~ 50–70% HS reduction (Supplemental Fig. 1), consistent with GCX degradation under disturbed flow conditions (~50%) [31, 40]. Higher concentrations yielded minimal additional degradation, whereas lower concentrations were insufficient to model pathological GCX shedding.

### RNA Sequencing

To determine the impact of flow exposure and HS degradation on a wide-range of indicators of pro-oxidant or pro-inflammatory endothelial activation, RNA-seq was applied to assess the presence, quantity, and dynamic expression of genes associated with either the endothelial redox state or endothelial inflammatory response. Four groups of samples were analyzed for gene expression using RNA-seq: (i) static conditioned, (ii) flow exposed, (iii) Hep III-treated and static conditioned, and (iv) Hep III-treated and flow exposed. Three samples from each group were sequenced. RNA-seq data was compiled into four gene sets: oxidant, antioxidant, pro-inflammatory, and anti-inflammatory.

The RNA-seq protocol was performed as follows. RNA from each sample was purified using a Qiagen RNeasy Plus Mini Kit following the manufacturer’s protocol. During RNA purification, RNA was also treated with Qiagen’s RNase-Free DNase set to remove any residual DNA. Libraries for RNA-seq were prepped using a NEBNext Ultra II Directional RNA Library Prep Kit with Poly(A) mRNA purification and indexed using NEBNext multiplex Oligos for Illumina, all from New England Biotech. To confirm RNA and library quality, both RNA and prepped libraries were analyzed using an Agilent 2100 Bioanalyzer with Agilent RNA 6000 and Agilent High Sensitivity DNA kits, respectively. Only samples with an RNA integrity above 8 on a scale from 1 to 10 were used. Sequencing was performed using an Illumina NextSeq500 instrument and a high output, 75x75 paired-end run, generating a total of 400 million reads for approximately 33 million reads per sample. Data generated from RNA-seq was analyzed in R to identify changes in gene expression.

Please refer to the Supplemental Materials and Methods document for more RNA-seq protocol details as provided by the Boston University Microarray and Sequencing Resource Core Facility, which provided substantial support for performing this work.

### Immunocytochemistry

Most samples processed for immunocytochemistry were fixed with 4% paraformaldehyde, while samples to be labeled for GCX components (as reported in the Supplemental Fig. 1) were fixed with 2% paraformaldehyde mixed with 0.1% glutaraldehyde. Fixation occurred for 20 minutes at room temperature. After fixation, cells were permeabilized with 0.2% Triton X-100 for 5 minutes at room temperature, with the exception of samples that did not require permeabilization because they would be stained for surface GCX (see Supplemental Fig. 1). Next, cells were primarily blocked with 5% goat serum, although for E-selectin the samples were blocked in 10% goat serum. Blocking was performed for 1 hour at room temperature. Following blocking, samples were incubated with their corresponding primary antibodies overnight at 4 °C at the following concentrations: HS (1:200; see Supplemental Fig. 1), KLF2 (1:500), ICAM-1 (1:500), E-selectin (10 μg/ml), Nrf2 (1:100), Nf-κb p65 (1:100). Most samples were incubated with the species appropriate Alexa Fluor 488-conjugated secondary antibody for 1 hour at room temperature at a 1:1,000 dilution. However, for E-selectin, cells were incubated in a biotinylated secondary antibody at a 1:1,000 dilution for 1 hour at room temperature, and then the cells were incubated with a tertiary streptavidin at a dilution of 1:1000 for signal amplification [40]. Following secondary or tertiary antibody incubation and subsequent phosphate buffered saline washes, samples were mounted using VectaShield Antifade Mounting Medium with 4′,6-diamidino-2-phenylindole (DAPI) and imaged using a Zeiss LSM 710, 800, or 880 confocal microscopes. HS (Supplemental Fig. 1), KLF2, ICAM-1, and E-selectin expression were quantified via mean fluorescent intensity (MFI) analysis. Nrf2 transcription factor activation was quantified by calculating the Pearson’s correlation coefficient between the respective transcription factor and DAPI fluorescent channels using the ImageJ Coloc 2 plug-in. Nf-κb activity was quantified via MFI analysis due to unrecognizable nuclear localization in all samples.

### Western Blotting

For Western blot analysis, samples were first washed twice in ice-cold phosphate buffered saline and then lysed using radioimmunoprecipitation assay (RIPA) buffer containing 150 mM sodium chloride (NaCl), 1% Triton X-100, 50 mM Tris base, 0.1% sodium dodecyl sulfate (SDS), 5 mM ethylenediaminetetraacetic acid (EDTA), 1 mM phenylmethylsuphonyl fluoride (PMSF), and Roche cOmplete EDTA-free protease inhibitor cocktail. To determine lysate protein concentration, a Pierce BCA (bicinchoninic acid) Protein Assay kit was used following the manufacturer’s protocol and measured using a NanoDrop One UV-Vis (ultraviolet–visible) spectrophotometer. For SDS polyacrylamide gel electrophoresis (SDS-PAGE), pre-determined volumes of lysate were incubated in BioRad 2 × Laemmli Sample Buffer at a 1:1 ratio and heated at 95 °C for 5 min. Samples were then loaded in Any kD Mini-Protean Stain-Free or 10% polyacrylamide gels and run at 120 V. Following SDS-PAGE, proteins were transferred onto polyvinylidene difluoride (PVDF) membranes via wet transfer. Membranes were blocked using 5% milk for 1 hour at room temperature, followed by primary antibody incubation overnight at 4 °C on a rocker at the following concentrations: ICAM-1 (1:1,000), Nox2 (1:750), Nox4 (1:750), and β -actin (1:3,000). Secondary antibody incubations were performed for 1 hour at room temperature on a rocker utilizing the appropriate species-specific horseradish peroxidase-conjugated antibody at 1:3,000 for Nox2 and Nox4, 1:2,000 for ICAM-1, or 1:10,000 for β -actin. Chemiluminescent detection was performed using the BioRad Clarity enhanced chemiluminescence reagents and imaged with a BioRad ChemiDoc Touch Imaging System. Densitometry of each protein was performed using ImageJ and presented as averages normalized to β -actin signal.

### Reactive Oxygen Species Assays

Two fluorescence-based ROS assays, dihydroethidium (DHE), to identify mainly superoxide [41], and 6-chloromethyl-2’, 7’-dichlorodihydrofluorescein diacetate (H_2_DCFDA), a more general indicator of ROS including hydrogen peroxide (H_2_O_2_) [42], were performed to identify ROS production. DHE and H2DCFDA were selected for their complementary detection of superoxide and general ROS, and because they are widely validated probes that we expect to provide sensitive, reliable readouts of oxidative stress in HAECs. For both assays, cells were first washed twice with warm Hank’s buffered saline solution (HBSS). Cells were then incubated in DHE or H_2_DCFDA diluted in HBSS at a concentration of 10 μM for 30 minutes at 37 °C and 5% CO_2_. At the conclusion of the 30-minute incubation, the cells were washed twice with HBSS and imaged using a Zeiss AxioObserver widefield microscope equipped with an incubator set at 37 °C. During imaging, cells were maintained in HBSS. For each sample, nine images were taken. The degree of ROS presence was determined by performing MFI analysis for each image using ImageJ.

### Statistical Analysis

For RNA-seq data, differential RNA expression was assessed using the Wald test implemented in the DESeq2 R package. Correction for multiple hypothesis testing was accomplished using the Benjamini-Hochberg false discovery rate (FDR). To identify changes in expression of entire gene sets, Gene Set Enrichment Analysis (version 2.2.1) [25] was used. Specifically, groups of the oxidant, antioxidant, pro-inflammatory, and anti-inflammatory genes containing 3, 39, 58, and 22 genes, respectively, were analyzed. All analyses were performed using the R environment for statistical computing (version 3.6.0).

Data pertaining to fluorescent imaging (HS (in Supplemental Fig. 1), KLF2, ICAM-1, E-selectin, Nrf2, Nf-κb, ROS probes) and Western blotting were analyzed using one-way ANOVAs with post hoc Tukey’s multiple comparison tests with an alpha value of 0.05. Prior to analysis, data was validated for normal distributions using the Shapiro Wilk test. Additionally, with the exception of RNA-seq data, data was normalized to control static groups in each experiment to eliminate any bias introduced by inter-experiment differences such as cell passage number. For protein quantification via MFI, a single data point was represented by the average of 1 to 2 individual slides with 3-4 images taken per slide to account for variability in expression depending on the region imaged.

## Results

### Gene-level Regulation of Inflammation: Flow Enhances Anti-Inflammatory Gene Expression, While HS Degradation Upregulates Pro-Inflammatory Genes

Numerous studies suggest that an increase in pro-inflammatory proteins, such as adhesion molecules, combined with a decrease in anti-inflammatory molecules, leads to endothelial activation [22, 43]. However, the relationship between flow, HS degradation, and inflammatory phenotype remains an active area of investigation. To contribute to this investigation, our initial approach was to perform RNA-seq to examine gene-level changes associated with flow and HS degradation, laying the groundwork for subsequent protein-level analysis in this study.

**Fig. 2:**
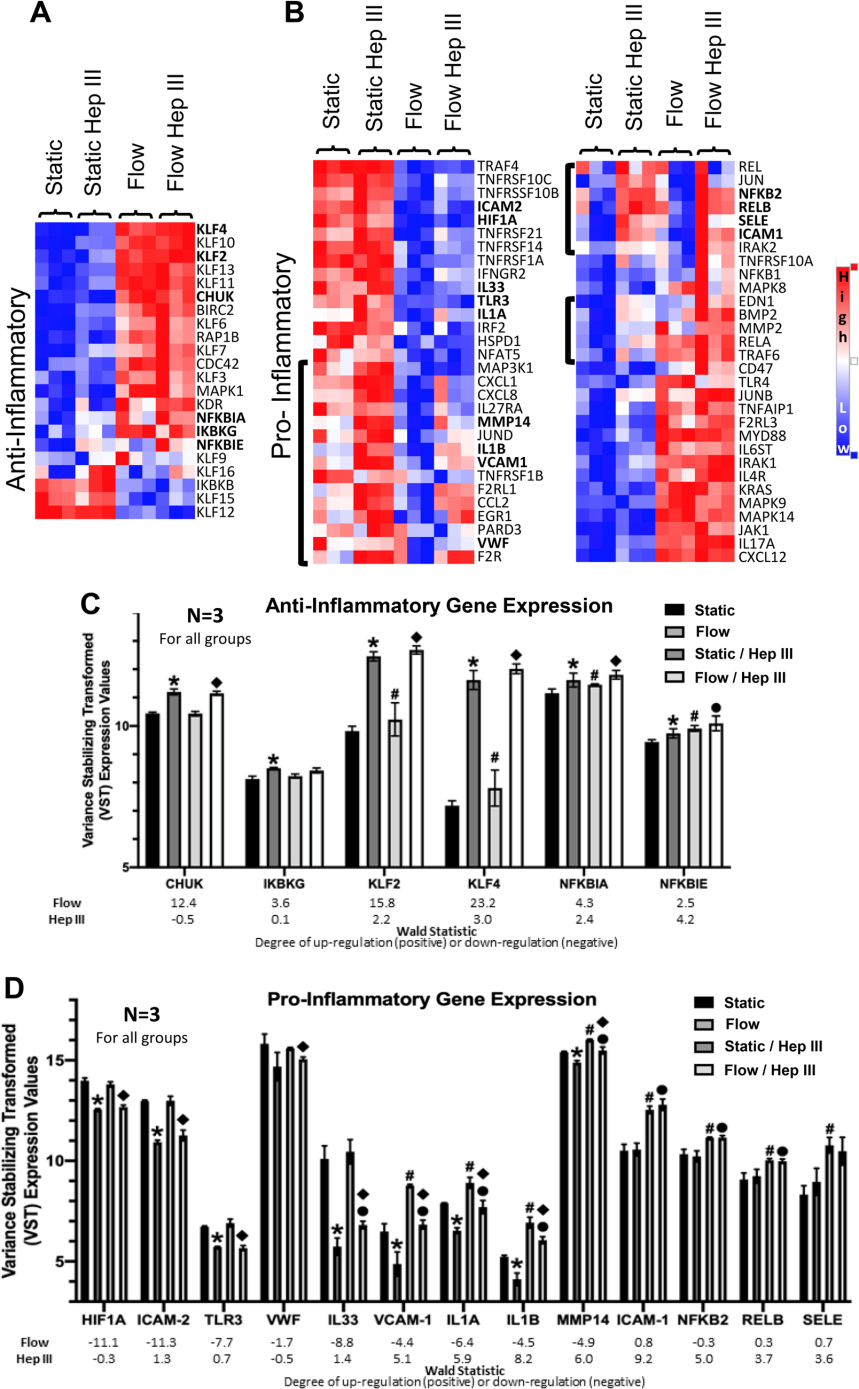
*Flow upregulates anti-inflammatory genes while HS degradation upregulates pro-inflammatory genes*. **A**, **B** Heat maps of endothelial cell **A** pro- and **B** anti-inflammatory genes in static, flow, static / Hep III, and flow / Hep III samples. Red indicates high levels of expression while blue indicates low levels of expression. Brackets to the left of the heat maps indicate gene groupings affected by HS degradation. **C**, **D** Plots show a subset of **C** anti- and **D** pro-inflammatory genes particularly implicated in endothelial cell function, highlighting a shift towards an anti-inflammatory phenotype after flow exposure, and a shift towards pro-inflammatory phenotype after HS degradation. Wald statistics, which indicate the degree of up-regulation (positive) or down-regulation (negative), are also provided. N = 3 for all groups. * = p < 0.05 between static and flow; # = p < 0.05 between static and Hep III static; ● = p < 0.05 between flow and flow / Hep III; ◆ = p < 0.05 between static / Hep III and flow / Hep III

Gene Set Enrichment Analysis, which examines differences in gene set expression between experimental groups, revealed that flow exposure, compared to static conditions, significantly up-regulated anti-inflammatory genes (p < 0.001; FDR q < 0.001) (Fig. 2a and c, Tables 1, 2). Specifically, flow increased the expression of 15 out of 22 investigated anti-inflammatory genes, including those associated with Nf-κb inhibition (CHUK, IKBKG, NFKBIA, and NFKBIE) and members of the KLF transcription factor family, with KLF2 and KLF4 being the strongest contributors to anti-inflammatory endothelial function (Fig. 2c). In contrast, of the 60 pro-inflammatory genes linked to activation, 30 exhibited significantly decreased expression following flow exposure. These included adhesion molecules (ICAM-2, VCAM-1), the transcription factor HIF1A, cytokines (IL1A, IL1B, IL33), the immune receptor TLR3, the glycoprotein VWF, and the endopeptidase MMP14 (Fig. 2b and c; Table 2). However, 26 of the 60 pro-inflammatory genes were significantly up-regulated (Table 2).

**Table 1:** Statistical parameters from Gene Set Expression Analysis for pro- and anti-inflammatory genes

	Pro–Inflammatory				Anti–Inflammatory			
Condition/Treatment	ES	NES	p–value	FDR q–value	ES	NES	p–value	FDR q–value
Flow	–0.32	–1.04	0.391	0.400	0.70	1.95	0.002	0.001
Hep III	0.73	2.43	< 0.001	< 0.001	0.67	1.83	0.003	0.001
Static vs Flow	–0.34	–1.14	0.230	0.557	0.73	2.03	< 0.001	< 0.001
Static vs Static/Hep III	0.75	2.45	< 0.001	< 0.001	0.73	1.94	< 0.001	< 0.001
Flow vs Flow/Hep III	0.72	2.49	< 0.001	< 0.001	0.85	1.40	0.227	0.215
Static/Hep III vs Flow/Hep III	–0.33	–1.07	0.337	0.359	0.70	1.95	< 0.001	< 0.001

Enrichment scores (ES) and normalized enrichment scores (NES) indicate the degree of gene set skewness, reflecting up-regulation (positive values) or down-regulation (negative values) in response to specific conditions/treatments. P-values assess statistical significance, while FDR q-values estimate the likelihood of false-positive results. Data includes both treatment effects (e.g., Flow, Hep III) and comparisons between experimental groups (e.g., static vs. flow).

**Table 2:** Flow-regulated inflammatory genes

Flow							
Pro–Inflammatory				Anti–Inflammatory			
Up–Regulated		Down–Regulated		Up–Regulated		Down–Regulated	
Gene	Wald Statistic	Gene	Wald Statistic	Gene	Wald Statistic	Gene	Wald Statistic
CXCL12	17.6	TRAF4	–21.9	KLF4	23.2	KLF12	–13.5
IL17RA	16.9	TNFRSF10C	–12.4	KLF10	17.4	KLF15	–7.1
JAK1	14.0	TNFRSF10B	–12.1	KLF2	15.8	IKBKB	–5.9
MAPK14	13.8	ICAM2	–11.3	KLF13	14.3		
MAPK9	10.1	HIF1A	–11.1	KLF11	14.0		
KRAS	9.9	TNFRSF21	–10.4	CHUK	12.4		
IL4R	9.7	TNFRSF14	–9.6	BIRC2	11.7		
IRAK1	8.7	TNFRSF1A	–9.6	KLF6	11.6		
IL6ST	8.7	IFNGR2	–9.1	KLF7	10.6		
MYD88	8.5	IL33	–8.8	KLF3	7.7		
F2RL3	8.2	TLR3	–7.7	KDR	5.4		
TNFAIP1	6.9	IL1A	–6.4	NFKBIA	4.3		
JUNB	6.8	IRF2	–6.3	IKBKG	3.6		
TLR4	5.9	HSPD1	–6.3	NFKBIE	2.5		
TLR4	5.9	NFAT5	–6.2	KLF9	1.6		
CD47	5.5	MAP3K1	–6.1				
TRAF6	5.4	CXCL1	–5.5				
RELA	5.3	CXCL8	–5.3				
MMP2	4.2	IL27RA	–5.1				
BMP2	4.2	MMP14	–4.9				
EDN1	4.2	JUND	–4.8				
MAPK8	3.4	IL1B	–4.5				
NFKB1	3.4	VCAM1	–4.4				
NFKB1	3.4	TNFRSF1B	–4.4				
TNFRSF10A	1.9	F2RL1	–4.2				
IRAK2	1.0	CCL2	–3.4				
		EGR1	–2.7				
		PARD3	–2.1				
		VWF	–1.7				
		F2R	–1.1				

Wald statistics quantify the extent of gene regulation, with positive values indicating up-regulation and negative values indicating down-regulation. A Wald statistic of ≥ 1 or ≤ − 1 denotes significant gene expression changes.

HS was also found to regulate gene expression related to pro-inflammatory endothelial cell activation. Specifically, Gene Set Enrichment Analysis identified an up-regulation in pro-inflammatory genes after HS degradation (p < 0.001; FDR q < 0.001), correlating with the increased expression of 42 out of the 60 investigated genes (Table 1). These included the adhesion molecules ICAM-1, SELE, and VCAM-1; the cytokines IL1A and IL1B; MMP14; and the Nf-κb subunits NFKB2 (p52/100) and RELB (Fig. 2d, Table 3). The observed increase in pro-inflammatory gene expression after HS degradation occurred in both static (static vs. static/Hep III) and flow (flow vs. flow/Hep III) conditions (Table 1). These increases were also interestingly accompanied by a significant increase in anti-inflammatory gene expression (p = 0.003; FDR q = 0.001), suggesting a complex relationship between HS degradation and inflammatory state.

**Table 3:** Heparan sulfate (HS) regulated inflammatory genes

Heparan Sulfate							
Pro–Inflammatory				Anti–Inflammatory			
Up–Regulated		Down–Regulated		Up–Regulated		Down–Regulated	
Gene	Wald Statistic	Gene	Wald Statistic	Gene	Wald Statistic	Gene	Wald Statistic
ICAM1	9.2	TNFRSF1B	–3.0	KLF7	5.0	KLF12	–1.5
IL1B	8.2	TLR4	–2.3	NFKBIE	4.2		
F2RL1	7.9	MAPK14	–1.4	KLF4	3.0		
EDN1	7.1	HSPD1	–1.2	LDLR	2.9		
MMP14	6.0	MAPK9	–1.1	KLF10	2.9		
IL1A	5.9			KLF6	2.9		
CCL2	5.7			KLF16	2.8		
TNFRSF21	5.1			BIRC2	2.7		
VCAM1	5.1			NFKBIA	2.4		
BMP2	5.1			KLF2	2.2		
NFKB2	5.0			KDR	1.3		
JUNB	4.9			KLF11	1.0		
CXCL8	4.8						
IFNGR2	4.3						
IL17RA	4.1						
EGR1	4.1						
F2R	4.1						
TNFRSF10B	4.0						
RELB	3.7						
SELE	3.6						
JUN	3.5						
CXCL1	3.5						
IRAK2	3.2						
IL4R	3.2						
TNFRSF10A	3.0						
CXCL12	2.8						
TNFRSF10C	2.7						
IRAK1	2.6						
JUND	2.6						
RELA	2.5						
NFKB1	2.4						
NFKB1	2.4						
CD47	2.3						
MMP2	2.1						
TRAF6	1.9						
TNFAIP1	1.5						
IL33	1.4						
F2RL3	1.3						
TNFRSF14	1.3						
ICAM2	1.3						
REL	1.1						
IL27RA	1.0						

Wald statistics quantify gene regulation, with positive values indicating up-regulation and negative values indicating down-regulation. A Wald statistic of ≥ 1 or ≤ − 1 denotes significant changes in gene expression.

### Protein-Level Regulation of Inflammation: Flow Enhances Anti-Inflammatory Proteins, While HS Degradation Increases Pro-Inflammatory Proteins

Immunocytochemistry and Western blotting were utilized to examine the effects of flow and HS degradation on anti-inflammatory (KLF2) and inflammatory (ICAM-1, E-selectin) protein expression.

**Fig. 3:**
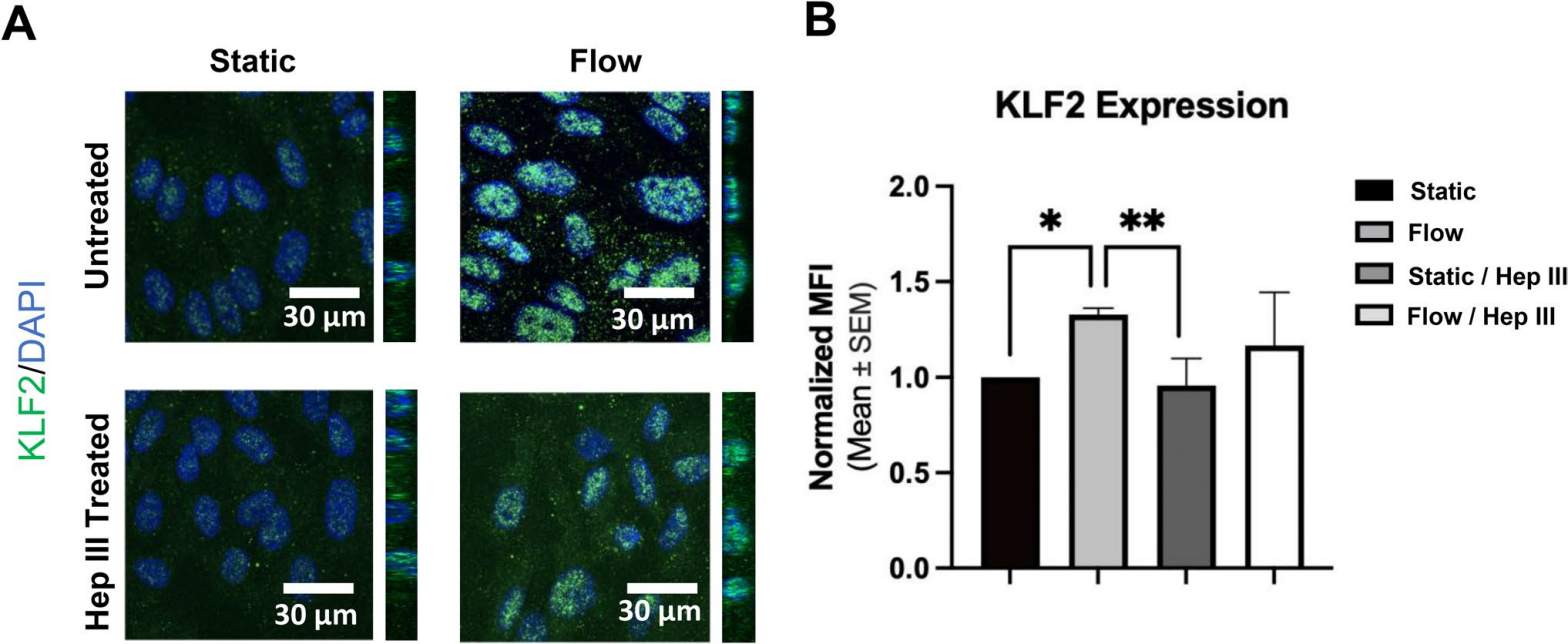
*Up-regulation of KLF2 gene expression by flow translates to increased KLF2 transcription factor protein levels. HS degradation seems to dampen the effect of flow on KLF2 protein expression*. **A** 63x immunocytochemistry images show the anti-inflammatory marker KLF2 (green) and DAPI (blue). **B** Normalized mean fluorescent intensity (MFI) of KLF2 in static, flow, static / Hep III, and flow / Hep III conditions indicates flow significantly increases KLF2 expression compared to static conditions, whereas Hep III treatment limits this flow-induced up-regulation. Sample size: n = 5 per group. Statistical significance is indicated by * = p < 0.05 and ** = p < 0.01

Flow led to a 32.8% increase in KLF2 MFI compared to static conditions (p < 0.05; Fig. 3a and b). Hep III treatment dampened this flow-induced effect, resulting in a non-significant 16.8% increase in KLF2 MFI under flow compared to static conditions when Hep III was present. Additionally, a 2.1% increase was observed when comparing static/Hep III to flow/Hep III conditions (Fig. 3a and b). In contrast, RNA-seq data (Fig. 2C) show that flow induces KLF2 gene up-regulation relative to static conditions in both the absence and presence of Hep III. However, this transcriptional up-regulation does not fully translate to protein-level expression. Flow promotes KLF2 protein expression only in the absence of Hep III, and even then, the increase is modest compared to the gene-level response, suggesting post-transcriptional regulatory bottlenecks. When Hep III is present, flow induces only a subtle, non-significant increase in KLF2 protein compared to static conditions. These findings indicate that Hep III selectively blunts flow-mediated up-regulation of KLF2 protein, an effect not observed at the transcriptional level. This discrepancy highlights the likely involvement of additional post-transcriptional or regulatory mechanisms that modulate KLF2 expression. The adhesion molecules ICAM-1 and E-selectin were examined to assess how flow and HS degradation influence pro-inflammatory protein expression.

**Fig. 4:**
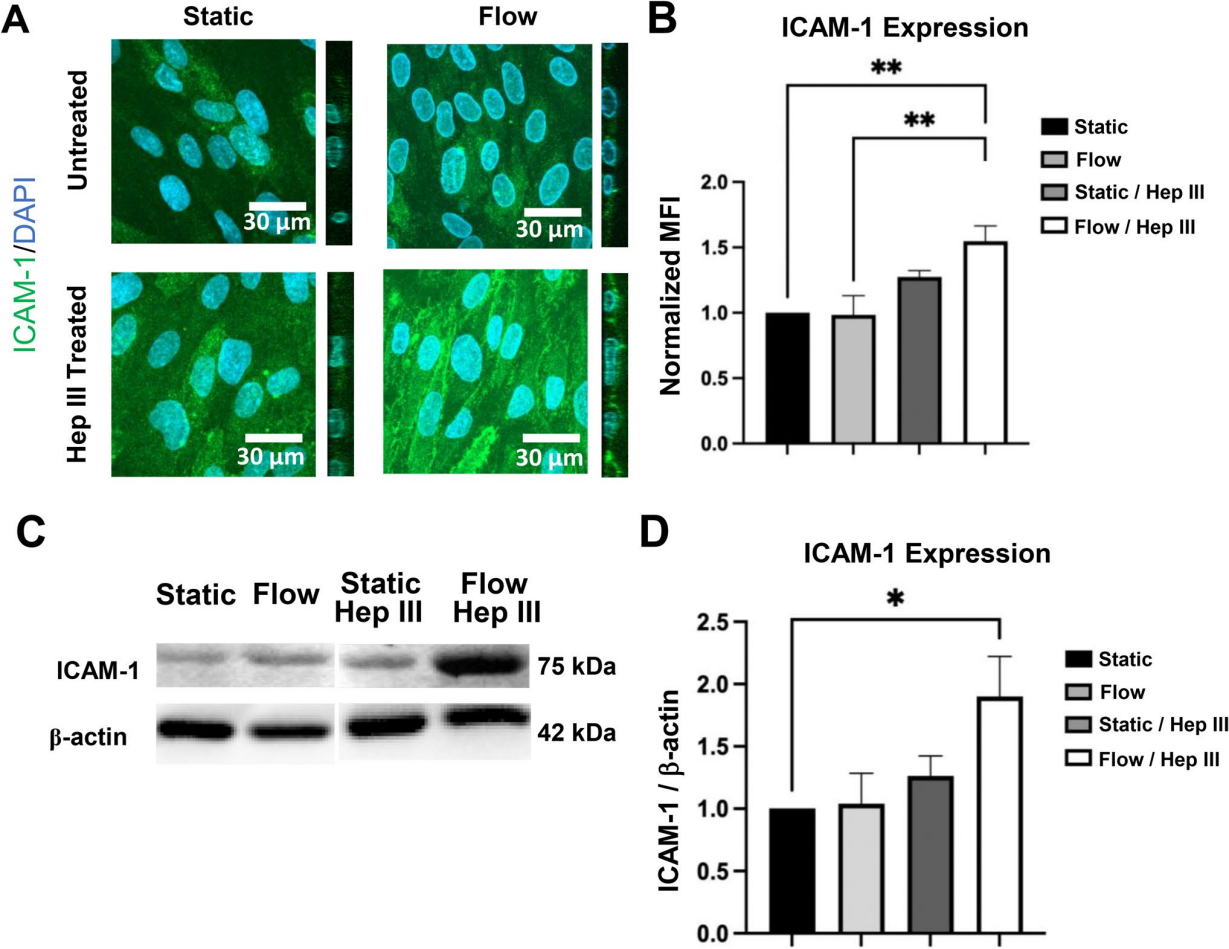
*Similar to RNA-seq data showing no impact of flow on ICAM-1 gene expression, protein analysis confirms the same trend. However, while RNA-seq data indicates Hep III significantly upregulates ICAM-1 in both static and flow conditions, protein analysis reveals a weaker trend in the same direction; significant ICAM-1 up-regulation requires the combined presence of Hep III and flow*. **A** 63x immunocytochemistry images of the inflammatory marker ICAM-1 (green) with DAPI (blue) in static, flow, static/Hep III, and flow/Hep III conditions. **B** Quantification of mean fluorescent intensity (MFI) from static, flow, static/Hep III, and flow/Hep III conditions demonstrates significantly elevated ICAM-1 expression in flow/Hep III samples compared to static and static/Hep III conditions. **C** Western blot analysis of ICAM-1, with **D** quantification showing significantly higher ICAM-1 levels in flow/Hep III conditions compared to static conditions. Sample size: n = 5 per group. Statistical significance is indicated by * = p < 0.05 and ** = p < 0.01

Immunocytochemistry showed that ICAM-1 expression in static conditions remained unaffected by Hep III. Flow alone did not alter ICAM-1 levels compared to static conditions, but the addition of Hep III under flow significantly increased ICAM-1 expression by 54.8% compared to static conditions (p < 0.01; Fig. 4a and b). Flow/Hep III conditions also increased ICAM-1 expression by 57.5% relative to flow without Hep III, with statistical significance (p < 0.01; Fig. 4a and b). ICAM-1 levels in static/Hep III samples appeared elevated relative to both static and flow conditions lacking Hep III yet remained below those observed in flow/Hep III samples (Fig. 4a and b). These differences, however, did not reach statistical significance. Western blot analysis further assessed ICAM-1 protein expression across static, flow, static/Hep III, and flow/Hep III conditions. Consistent with immunocytochemistry, Western blot data showed a 90.2% increase in ICAM-1 expression in flow/Hep III samples compared to static (p < 0.05) and an 82.9% increase compared to flow (approaching statistical significance; p = 0.0552) (Fig. 4c and d). ICAM-1 expression under static/Hep III conditions showed a modest increase compared to static and flow samples without Hep III but was still lower than the levels seen in flow/Hep III (Fig. 4c and d). None of these comparisons reached statistical significance. Recall that RNA-seq data show that flow alone has no significant effect on ICAM-1 gene expression compared to static conditions, while HS degradation via Hep III increases ICAM-1 transcription under both static and flow conditions (Fig. 2d). However, immunocytochemistry and Western blot results only partially align with this trend. ICAM-1 protein elevation is observed exclusively under flow/Hep III conditions, but not in static/Hep III, despite comparable gene-level up-regulation. This discrepancy highlights a disconnect between transcriptional and protein-level responses. Several factors may contribute to this misalignment. First, ICAM-1 protein stability and detectability may depend on post-translational modifications such as glycosylation, which could be limited under static conditions, restricting protein accumulation or surface presentation. Second, flow may enhance baseline HS expression or organization, making its enzymatic degradation by Hep III more impactful, potentially amplifying inflammatory signaling and ICAM-1 protein induction. Further investigation is needed to clarify the interplay between flow, HS structure, and post-transcriptional regulation in modulating ICAM-1 expression.

**Fig. 5:**
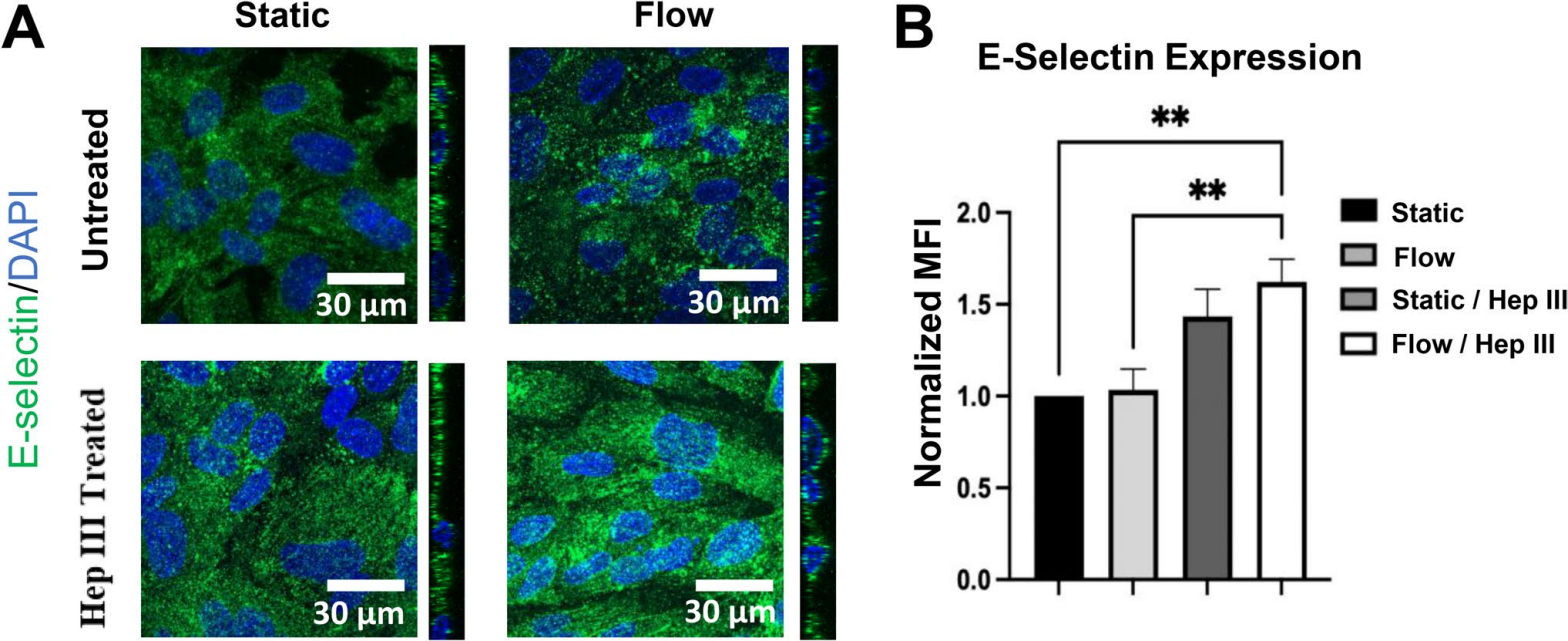
*Protein analysis shows no impact of flow on E-selectin levels. Additionally, E-selectin level is up-regulated with Hep III treatment, most significantly under flow conditions*. **A** 63x immunocytochemistry images of the inflammatory marker E-selectin (green) with DAPI (blue) in static, flow, static/Hep III, and flow/Hep III conditions. **B** Quantification of MFI from static, flow, static/Hep III, and flow/Hep III conditions. Sample size: n = 5 per group. Statistical significance is indicated by ** = p < 0.01

For E-selectin, flow/Hep III showed the most pronounced increase, with immunocytochemistry indicating a 62.2% and 56.8% elevation compared to static and flow conditions, respectively (p < 0.01; Fig. 5a and b). Static/Hep III also showed elevated E-selectin levels relative to static and flow conditions, though the increase was not statistically significant. These findings demonstrate partial alignment between RNA-seq (Fig. 2d) and protein-level data. Unlike ICAM-1, E-selectin protein expression more closely mirrors gene-level trends, although RNA-seq shows peak induction in static/Hep III while protein analysis highlights flow/Hep III as the highest condition. Importantly, both datasets consistently indicate that HS degradation contributes to E-selectin up-regulation.

### Gene-Level Regulation of Oxidative Stress: Flow Enhances Endothelial Antioxidant Activity, While HS Degradation has Minimal Influence

**Fig. 6:**
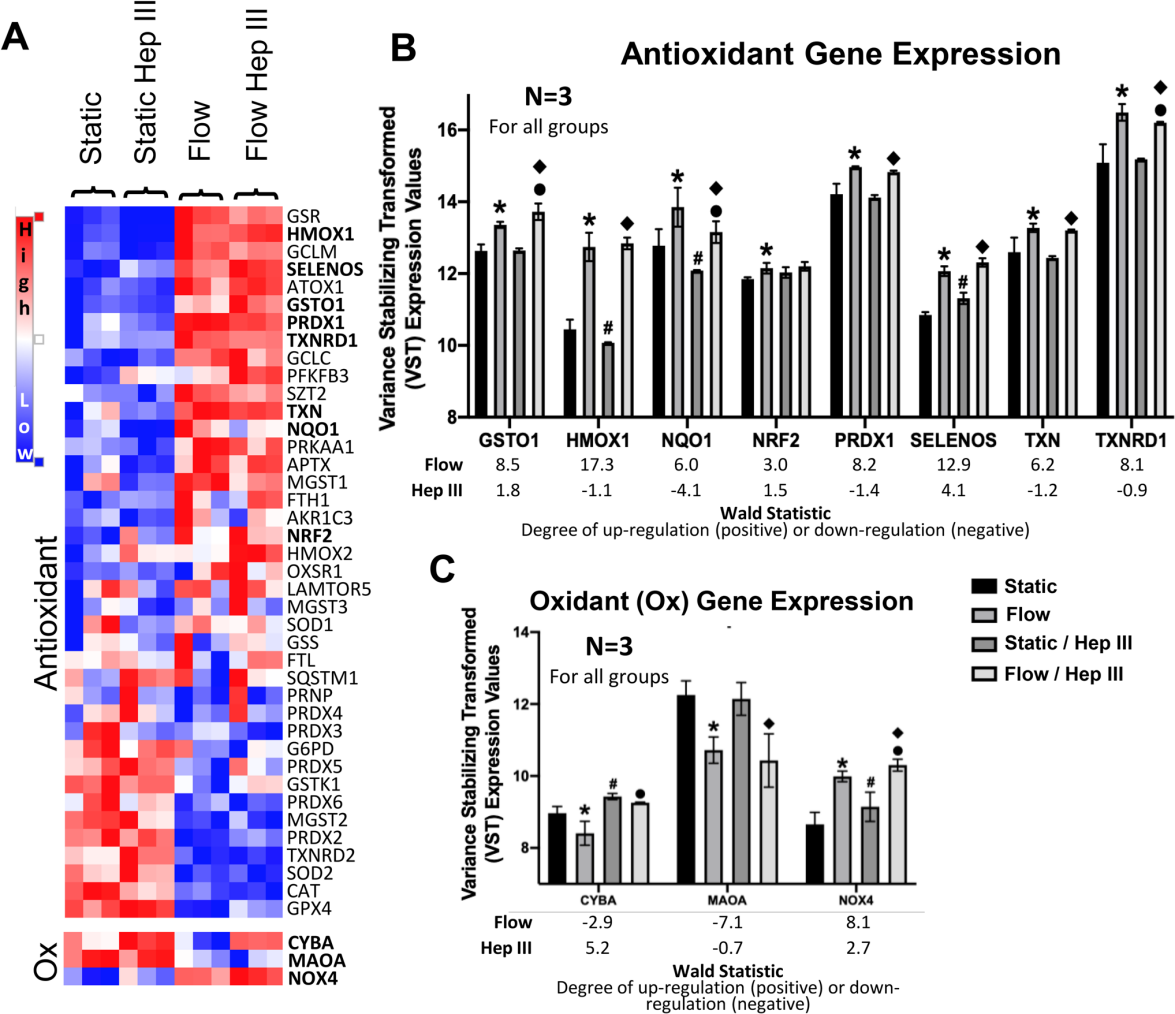
*Uniform flow enhances antioxidant gene expression, while HS degradation has no effect.* **A** Heat map of antioxidant (top) and oxidant (bottom) gene expression in static, Hep III-treated static, flow, and Hep III-treated flow conditions. Red indicates high gene expression levels, while blue represents low expression. The heat map demonstrates a positive skew in antioxidant gene expression under flow conditions, with no evident changes following HS degradation via Hep III. **B** Uniform flow significantly upregulates antioxidant genes, including *Prdx1, Nqo1, Txn, Nrf2, SELENOS, Txnrd1, Gsto1,* and *HO-1*, among others. Similar trends are observed in Hep III-treated samples, suggesting HS degradation does not affect antioxidant gene expression. Wald statistics indicate the degree of up-regulation (positive) or down-regulation (negative). **C** Flow significantly influences oxidant gene expression, with differential effects observed, including on *Cyba, Maoa,* and *Nox4*. Wald statistics are provided. N = 3 for all groups. * = p < 0.05 between static and uniform flow; # = p < 0.05 between static and Hep III static; ● = p < 0.05 between uniform flow and Hep III uniform flow; ◆ = p < 0.05 between Hep III static and Hep III uniform flow

Along with an up-regulation in inflammation, increased production of ROS such as hydrogen peroxide and superoxide occurs during endothelial activation associated with cardiovascular diseases such as atherosclerosis [14, 15]. RNA-seq was utilized to investigate the impact of flow and the HS component of the endothelial GCX on redox state while also identifying key genes involved. Gene Set Enrichment Analysis demonstrated a significant increase in antioxidant genes after exposure to flow (p = 0.002; FDR q = 0.019) (Fig. 6a and b; Table 4). Out of 41 antioxidant genes with significant expression (>1000 counts) that had previously been implicated in endothelial redox regulation, we observed increased expression in 23 of these genes after exposure to flow in non-Hep III conditions (Fig. 6a and b; Table 5). Specifically, significant increases in the expression of antioxidant genes including GSTO-1, HMOX-1, NQO1, NRF2, PRDX1, SELENOS, TXN, and TXNRD1 were observed to increase after flow exposure (Fig. 6b). With regard to oxidant genes, flow exposure reduced the expression of the oxidant genes CYBA, a Nox protein subunit, and MAOA while simultaneously increasing NOX4 expression (Fig. 6a and c). However, Gene Set Enrichment Analysis found no significant changes in oxidant gene expression after flow (p = 0.879; FDR q = 0.945) (Table 4). No significant expression of Nox2 was identified.

**Table 4:** Statistical parameters from Gene Set Expression Analysis for oxidant and antioxidant genes

	Oxidant				Antioxidant			
Condition / Treatment	ES	NES	p–value	FDR q–value	ES	NES	p–value	FDR q–value
Flow	0.39	0.64	0.879	0.945	0.50	1.55	0.002	0.019
Hep III	0.85	1.37	0.089	0.072	–0.27	–0.96	0.500	0.525
Static vs Flow	–0.39	–0.63	0.898	0.945	0.44	1.36	0.067	0.066
Static vs Static/Hep III	0.84	1.34	0.105	0.095	–0.29	–1.07	0.307	0.387
Flow vs Flow/Hep III	0.85	1.40	0.058	0.091	–0.28	–1.03	0.378	0.426
Static/Hep III vs Flow/Hep III	0.48	0.78	0.756	0.830	0.60	1.94	< 0.001	< 0.001

Enrichment scores (ES) and normalized enrichment scores (NES) assess gene set regulation. Positive values indicate up-regulation, while negative values suggest down-regulation. P-values denote statistical significance, while FDR q-values estimate the likelihood of false positives. Data captures the effects of specific treatments (e.g., Flow, Hep III) and comparisons between experimental groups (e.g., Static vs. Flow).

**Table 5:** Redox genes regulated by flow

Flow							
Oxidant				Antioxidant			
Up–Regulated		Down–Regulated		Up-Regulated		Down-Regulated	
Gene	Wald Statistic	Gene	Wald Statistic	Gene	Wald Statistic	Gene	Wald Statistic
NOX4	8.1	MAOA	–7.1	GSR	19.5	GPX4	–11.5
		CYBA	–2.9	HMOX1	17.3	SOD2	–8.8
				GCLM	14.8	TXNRD2	–7.9
				SELENOS	12.9	PRDX2	–7.4
				ATOX1	9.9	MGST2	–7.1
				GSTO1	8.5	PRDX6	–4.8
				PRDX1	8.2	GSTK1	–3.5
				TXNRD1	8.1	PRDX5	–3.3
				GCLC	7.2	G6PD	–2.9
				SZT2	6.9	PRDX3	–2.0
				TXN	6.2	PRDX4	–1.3
				NQO1	6.0	PRNP	–1.2
				PRKAA1	5.5		
				APTX	5.4		
				MGST1	3.9		
				FTH1	3.5		
				AKR1C3	3.5		
				NFE2L2	3.0		
				HMOX2	3.0		
				OXSR1	2.7		
				LAMTOR5	1.4		
				MGST3	1.3		
				SOD1	1.1		
				GSS	1.0		

Wald statistics quantify gene regulation, with positive values indicating up-regulation and negative values indicating down-regulation. Significant regulation is defined by Wald statistics of ≥ **1** (up-regulation) or ≤ **− 1** (down-regulation).

In contrast to the literature [34], Gene Set Enrichment Analysis failed to identify an impact of HS degradation on redox gene regulation (Table 4). This is evidenced by the observation that Hep III treatment in static conditions resulted in the decreased expression of 15 antioxidant genes but simultaneously up-regulated the expression of 13 antioxidant genes (Table 6). However, HS degradation did lead to an up-regulation of the oxidant genes NOX4 and CYBA (Fig. 6c), although Gene Set Enrichment Analysis identified no significant difference in expression when evaluating the entire oxidant gene set (p = 0.089; FDR q = 0.072) (Table 4).

**Table 6:** Redox genes regulated by HS

Heparan Sulfate							
Oxidant				Antioxidant			
Up–Regulated		Down–Regulated		Up–Regulated		Down–Regulated	
Gene	Wald Statistic	Gene	Wald Statistic	Gene	Wald Statistic	Gene	Wald Statistic
CYBA	5.2			HMOX2	4.2	NQO1	–4.1
NOX4	2.7			SELENOS	4.1	GSR	–3.4
				GPX4	3.7	GCLM	–3.2
				SQSTM1	2.2	PRDX3	–2.6
				PRDX5	2.0	MGST1	–2.4
				PRDX4	1.8	SZT2	–2.3
				GSTO1	1.8	AKR1C3	–2.2
				TXNRD2	1.7	PRDX6	–1.8
				PRNP	1.5	SOD1	–1.4
				NFE2L2	1.5	PRKAA1	–1.4
				PRDX2	1.0	PRDX1	–1.4
				SOD2	1.0	TXN	–1.2
				FTH1	1.0	HMOX1	–1.1
						APTX	–1.1

Wald statistics quantify gene regulation, with positive values indicating up-regulation and negative values indicating down-regulation. Significant regulation is defined by Wald statistics of ≥ 1 (up-regulation) or ≤ − 1 (down-regulation).

### Protein and Functional Regulation of Oxidative Stress: Flow Reduces ROS and Precursors, While HS Expression has Minimal Impact

To investigate how observed genetic changes from RNA-seq data translate into protein expression and functional outcomes, we employed immunocytochemistry and Western blotting, focusing on Nrf2, Nf-κb, Nox2, and Nox4. Additionally, fluorescent ROS probes were used to assess oxidative stress responses at a functional level.

**Fig. 7:**
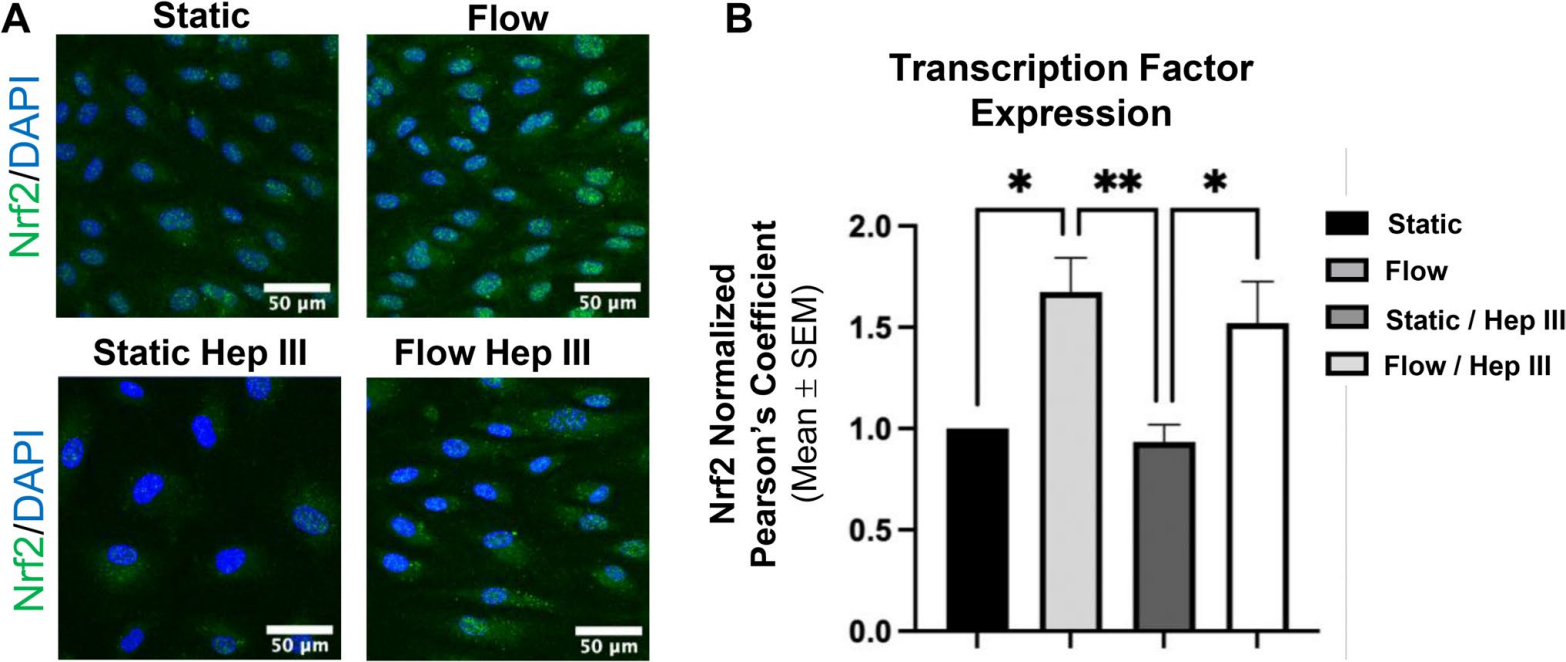
*Flow regulates endothelial phenotype by influencing transcription factor activation to promote an antioxidant response independent of HS expression*. **A** Immunocytochemistry images of the Nrf2 transcription factor, a key regulator of antioxidant defense and suppressor of oxidant agents such as Nox, show increased Nrf2 expression following flow exposure, both with and without Hep III. **B** Quantitative analysis of Nrf2 colocalization with cellular nuclei, serving as an indicator of activation, confirms a higher likelihood of activation under flow conditions. HS degradation does not interfere with this effect. Data for quantitative analysis was collected from static, flow, static/Hep III, and flow/Hep III conditions. Sample size: n = 5 per group. Statistical significance is indicated by * = p < 0.05 and ** = p < 0.01

We first examined Nrf2 and Nf-κb, key transcription factors that regulate oxidative stress in opposing ways. Nrf2, related to genes such as HO-1 and NQO1, promotes antioxidant defenses to neutralize ROS, while Nf-κb crosses over from pro-inflammatory pathways to exacerbate ROS levels. Immunocytochemistry revealed a significant increase in Nrf2 activation under flow, with a 67.4% rise compared to static (*p* < 0.05) and a 79.2% rise compared to static/Hep III (*p* < 0.01), as determined by its colocalization with endothelial nuclei (Fig. 7a and b). HS degradation via Hep III did not impair Nrf2 activation by flow. Conversely, Nf-κb-p65 expression showed no significant differences between static and flow conditions (Supplemental Fig. 2). Due to this lack of variability, experimental replicates were kept limited and nuclear colocalization (an indicator of activation) was not evaluated (Supplemental Fig. 2).

**Fig. 8:**
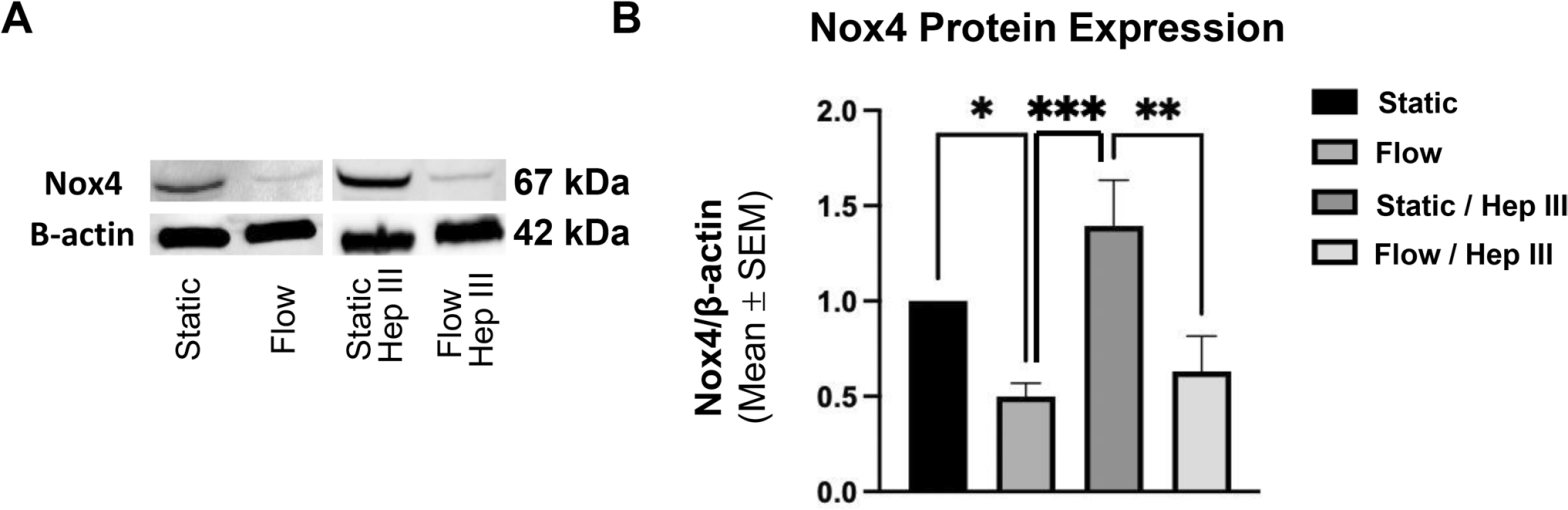
*Flow induces an antioxidant endothelial phenotype by reducing the expression of ROS precursor Nox4, independent of HS expression*. **A** Western blots show a visible reduction in Nox4 expression following flow exposure, which remains unchanged when HepIII is introduced. **B** Nox4 quantification was based on static (n = 7), flow (n = 7), static/Hep III (n = 5), and flow/Hep III (n = 5) conditions. Data demonstrates a statistically significant reduction in Nox4 expression after flow exposure, regardless of HS integrity, indicating that HS degradation does not influence the antioxidant response induced by flow. Statistical significance is indicated by * = p < 0.05, ** = p < 0.01, and *** = p < 0.001

Next, we examined the ROS precursors Nox2 and Nox4 to assess how flow and HS degradation influence redox state downstream of transcription factor activity. Western blot analysis revealed that flow significantly reduced Nox4 protein expression, with a 50.0% reduction compared to static conditions (*p* < 0.05) (Fig. 8a and b). This reduction was preserved under flow/Hep III, which showed a 54.8% decrease compared to static/Hep III conditions (*p* < 0.01) (Fig. 8a and b). Additionally, Hep III alone under static conditions led to a 39.5% increase in Nox4 expression relative to static, though this change was not significant (Fig. 8a and b). Flow/HepIII versus flow without HepIII were not statistically significantly different from each other either (Fig. 8a and b). Like Nox4, Nox2 expression decreased in flow conditions with and without HepIII, compared to static conditions with and without HepIII, and did so dramatically, reaching 10% of static levels (Supplemental Fig. 3). Minimal Nox2 experimental replicates were performed (2 to 3 replicates per group) because the results mirrored the Nox4 dataset. Together, the Nox2 and Nox4 results indicate that flow suppresses ROS-generating enzyme expression regardless of HepIII-induced HS degradation, aligning with the observed Nrf2 activation trends, and suggesting that the antioxidant response induced by flow is independent of HS integrity.

**Fig. 9:**
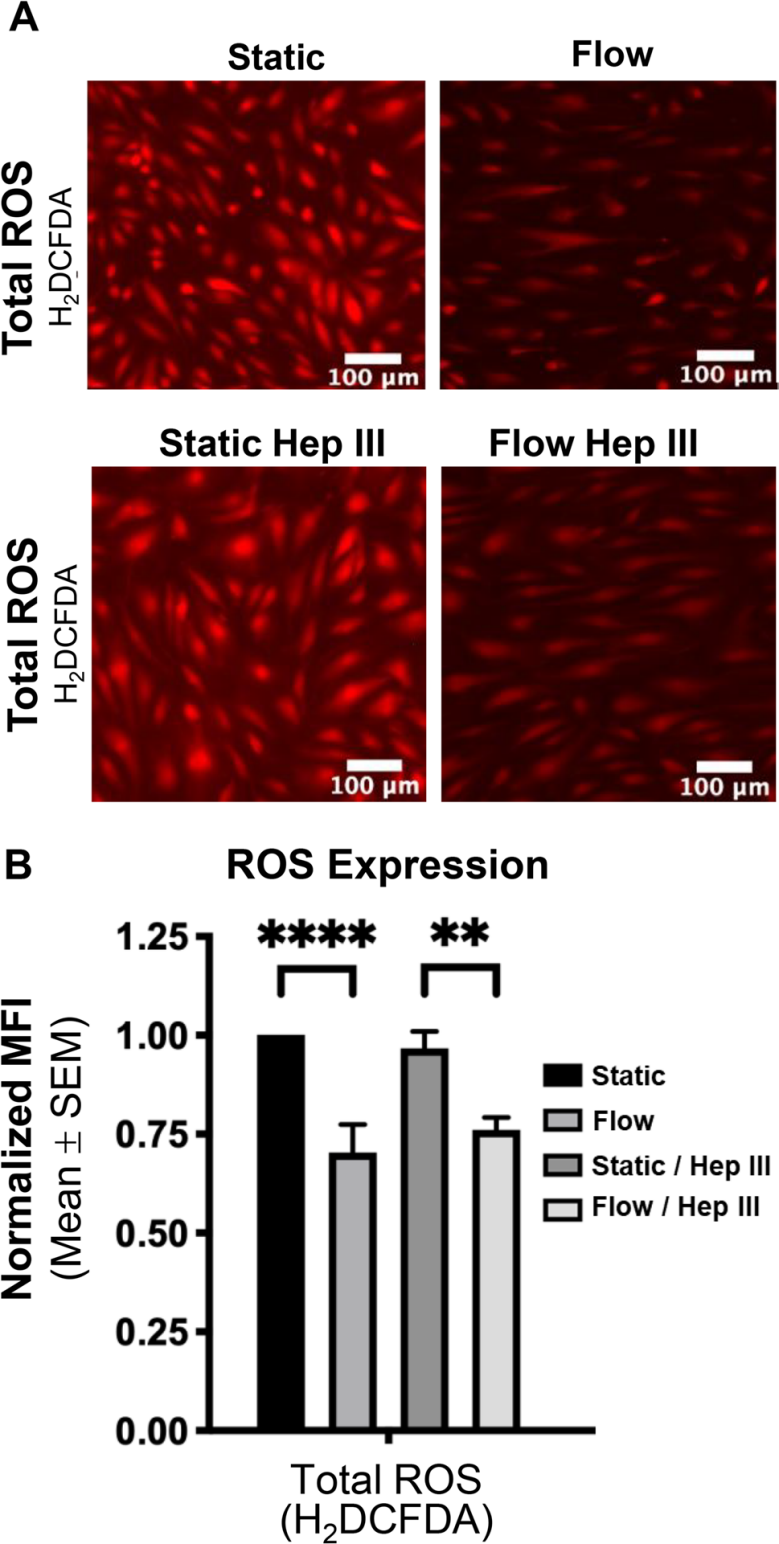
*Flow reduces ROS levels independently of HS expression.* **A** A fluorescent ROS assay using H2DCFDA demonstrates this effect, showing decreased fluorescence intensity under flow conditions. **B** Quantification of MFI from fluorescent images (n = 6 for all conditions) confirms the statistical significance of these findings, indicating a significant reduction in ROS following flow exposure, regardless of HS degradation. Statistical significance is denoted by ** = p < 0.01 and **** = p < 0.0001

Finally, we assessed ROS levels directly. Using the fluorescent ROS indicator H2DCFDA, we found that flow reduced total ROS levels by nearly 30% compared to static controls (Fig. 9a and b). Consistent with RNA-seq data, HS degradation via Hep III had no impact on ROS production (Fig. 9a and b). The flow-induced ROS reduction persisted regardless of HS presence, mirroring results from non-enzyme conditions. To further investigate oxidative mechanisms, we probed for superoxide, a ROS subtype. Flow and Hep III had no detectable effect on superoxide levels, as determined by the DHE probe (Supplemental Fig. 4).

Taken together with RNA-seq findings, these protein and functional results indicate that the antioxidant response following flow exposure is independent of the HS component of the GCX.

## Discussion

Endothelial activation, marked by pro-inflammatory signaling and elevated ROS production, is a central mechanism in cardiovascular disease progression [1–6]. In this study, we examined how flow at uniform physiological shear stress levels, and the structural integrity of the GCX, particularly the HS component, regulate endothelial inflammation and oxidative stress. Using a multimodal approach that included RNA-seq, immunocytochemistry, Western blotting, and functional imaging with fluorescent probes, we characterized both gene expression and protein-level changes in response to HS degradation and fluid shear stress. To our knowledge, this is the first study to integrate transcriptomic and protein-based analyses to broadly profile how HS degradation influences both inflammatory and redox regulatory pathways in endothelial cells. This comprehensive perspective revealed distinct signaling routes through which flow and HS independently shape endothelial phenotype.

Our results confirm that physiological flow promotes an anti-inflammatory phenotype in endothelial cells. Flow exposure led to decreased expression of pro-inflammatory genes and increased expression of anti-inflammatory markers at both the RNA and protein levels. These findings are consistent with prior studies, including work by Ajami *et al.* who demonstrated an anti-inflammatory effect of uniform, pulsatile flow, particularly when compared to disturbed, oscillatory flow [44]. More broadly, our results align with literature supporting the protective role of flow-derived shear stress in preventing pro-inflammatory endothelial activation [20, 45]. Therefore, our findings reaffirm the role of physiological flow in promoting vascular homeostasis. Our results also corroborate established HAEC transcriptomic datasets. Specifically, studies by Maurya et al. [46] and Meng et al. [47] demonstrated that uniform flow patterns (both steady and pulsatile forms) upregulate KLF2/KLF4 and downregulate pro-inflammatory cytokines and adhesion molecules, trends we observe consistently in our dataset. The strong overlap in differentially expressed genes between our study and these reports reinforces the validity of our flow model and confirms that 24-hour uniform shear stress reliably induces an anti-inflammatory endothelial phenotype.

Importantly, we identified a novel role for HS in suppressing pro-inflammatory endothelial activation. HS degradation led to increased expression of pro-inflammatory genes and elevated ICAM-1 and E-selectin protein levels, supporting its role in restraining inflammatory signaling. These findings are consistent with previous studies showing that HS degradation exacerbates endothelial activation, increasing adhesion molecule expression [32, 33] and activating pro-inflammatory transcription factors [32]. Notably, these effects occurred under both static and flow conditions, for the most part, supporting a model in which HS signaling modulates inflammation through a mechanism independent of flow-mediated mechanotransduction. This came as a surprise because prior research has primarily linked HS degradation to impaired mechanosensing [32, 33]. However, HS has importance beyond its mechanical function and is also recognized as an active signaling molecule [35–39]. In our study, this signaling capacity appears to operate in parallel with other regulatory mechanisms. We found that anti-inflammatory gene expression and KLF2 protein levels continued to increase in response to flow, even in the absence of HS. This suggests that flow exerts anti-inflammatory effects independently of HS, and together, these findings support a model in which HS and flow act through distinct but complementary pathways to regulate endothelial inflammation.

Our transcriptomic findings align with and extend previous RNA-seq studies examining HS degradation and flow-mediated endothelial responses. Regarding HS degradation, our results demonstrate agreement with studies by Richter et al. [48, 49], which similarly reported up-regulation of cytokines and markers of endothelial activation following enzymatic glycocalyx cleavage in endothelial cells. Although these studies were conducted in non‑HAEC contexts (human primary lung microvascular endothelial cells), the consistent inflammatory gene signatures across endothelial subtypes suggest a conserved role for HS in suppressing endothelial activation. Our findings extend this work by demonstrating that HS degradation promotes inflammation through mechanisms independent of flow-mediated mechanotransduction, as evidenced by sustained inflammatory gene up-regulation under both static and flow conditions.

We next investigated the endothelial redox state under flow and HS-degraded conditions. Previous studies have identified numerous genes that contribute to redox regulation, including oxidative mediators like the Nox family [50] and antioxidant genes such as HMOX-1 [51], NQO1 [52], and Nrf2 [53]. Our findings, informed primarily by high-throughput RNA-seq data, corroborate these earlier observations while also revealing widespread changes in redox activity, emphasizing the complexity of the endothelial oxidative stress response. In essence, and consistent with prior findings [27, 28], 24-hour exposure to physiological fluid shear stress significantly reduced ROS production and up-regulated antioxidant genes such as *HMOX1*, *NQO1*, and *Nrf2*, while downregulating oxidant proteins Nox2 and Nox4. These data support the notion that prolonged flow induces an antioxidant phenotype [27, 28]. Interestingly, our findings also align with earlier reports of ROS increases [22–24] because the increase occurred in short flow exposure time frames (approximately 30 minutes to 6 hours) preceding an eventual ROS decline [27, 54], suggesting a biphasic response that transitions from oxidative to antioxidant signaling over time. It is possible that had we captured redox readouts at earlier times, we may have observed this transient oxidative phase. With this said, the insights of our study, all taken together, warrant continued investigation using high-throughput platforms, not only for transcriptomic analysis (e.g., RNA-seq) and proteomic profiling (e.g., mass spectrometry, multiplex assays, protein microarrays), but also through live-cell imaging approaches that enable real-time visualization of redox fluctuations, subcellular localization, and dynamic signaling events over time.

We also looked into the role of GCX in regulating oxidative stress. While earlier work by Kumagai et al. reported that HS and sialic acid degradation elevated ROS levels in ex vivo vascular segments [34], we observed no such effect in isolated endothelial cells. This discrepancy may be due to cellular context. Kumagai et al.'s increased ROS signals were prominent in vessel layers containing smooth muscle and supportive vascular cells [34], whereas our study focused exclusively on endothelial responses. This distinction suggests that HS degradation may influence ROS production in a multicellular vascular environment but not directly within endothelial cells themselves. Further research is required to test this hypothesis.

The absence of detectable ROS regulation by HS may also reflect the inherent challenges of measuring ROS molecules. ROS are highly transient, can be converted into secondary oxidants such as peroxynitrite, and are often compartmentalized within cells, making them difficult to capture with standard assays. These challenges in ROS detection were mitigated by using H2DCFDA and DHE, complementary and widely validated probes that provide sensitive, reliable readouts of general and superoxide ROS. Indicators of permanent oxidative injury to proteins, lipids or nucleic acids, such as 4‑hydroxynonenal (HNE) adducts and thiobarbituric acid reactive substances (TBARS) for lipid peroxidation, or carbonyl modifications for protein oxidation, could, in principle, offer further insight. However, we carefully examined our RNA-seq dataset for evidence of sustained oxidative injury following HS degradation. Although RNA-seq does not directly measure oxidative adducts, it robustly captures the transcriptional consequences of permanent ROS damage. These include activation of canonical pathways such as microsomal GSTs involved in HNE and acrolein conjugation (MGST1/2/3) [55, 56], lipid hydroperoxide repair enzymes (GPX4) [57], protein peroxidation repair systems (PRDX1/3/4/5/6) [58], thiol-recycling and carbonyl-damage response enzymes (GSR, TXN, TXNRD1) [59], and Nrf2-regulated oxidative-stress markers (HMOX1, NQO1, GCLC, GCLM, GSS, SOD1, and CAT) [60]. These gene families were directly measured, yet none were significantly up-regulated under static or flow conditions after HS degradation. The collective evidence strongly argues that HS degradation did not impose detectable permanent oxidative injury. Thus, while additional biochemical assays could theoretically provide complementary information, our data strongly support the absence of transcriptional signatures consistent with sustained oxidative injury. Further adduct-based assays would be unlikely to alter the biological interpretation.

**Fig. 10:**
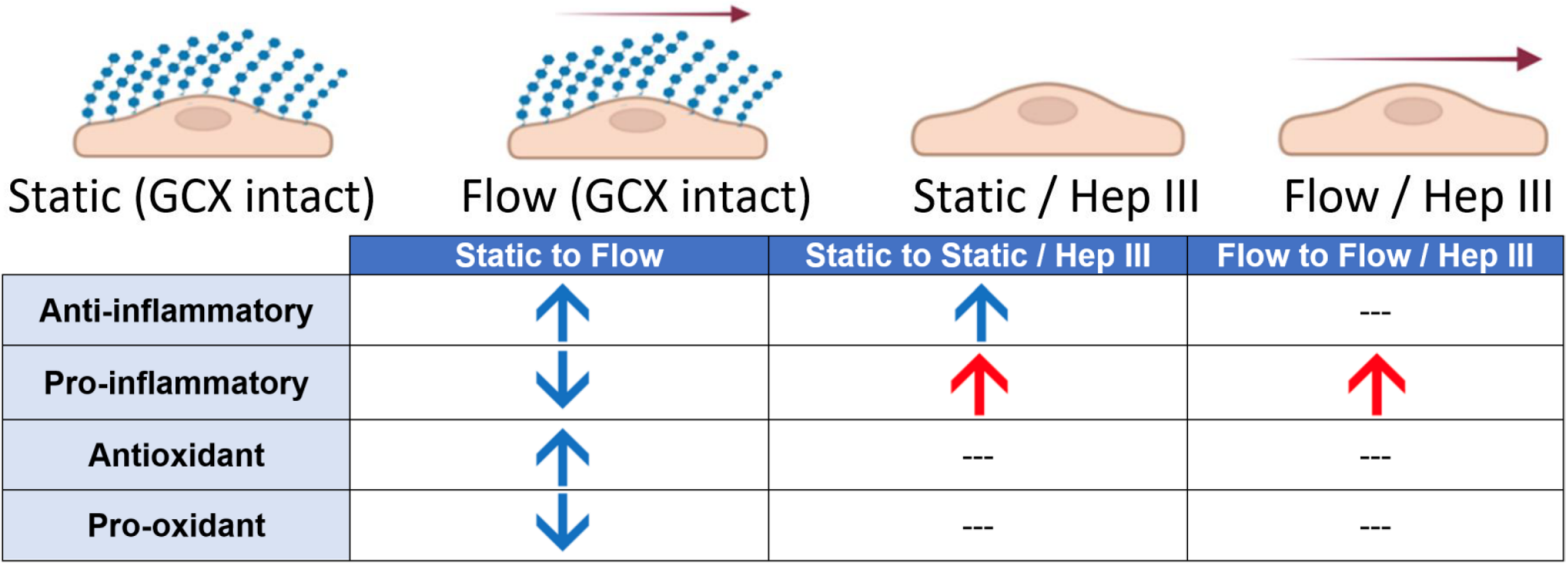
*Summary of endothelial activation trends across experimental conditions (This figure was created using BioRender.com).* Red arrows indicate conditions that promote endothelial activation (increased inflammation and oxidative stress), while blue arrows denote conditions that deter endothelial activation (reduced inflammation and oxidative stress). A dash (—) represents an ambiguous or unobserved trend

Taken together, these findings indicate a detrimental role of HS degradation in endothelial activation, marked by the up-regulation of pro-inflammatory molecules, and highlight the beneficial effects of fluid shear stress in promoting an anti-inflammatory and antioxidant endothelial state (Fig. 10). We acknowledge that our endothelial monoculture model omits local signaling interactions with smooth muscle cells, perivascular, and immune components, all of which are known to modulate inflammatory and redox pathways. Nonetheless, this monoculture design provided mechanistic isolation of endothelial responses, which was the central focus of this study. Future work will incorporate co-culture or multicellular models to capture these additional influences. With this said, the present study’s modest increase in anti‑inflammatory gene expression following HS removal indicates a nuanced interplay between HS signaling and inflammation. In contrast, flow-induced ROS regulation occurred independently of HS expression, diverging from our original hypothesis (Fig. 10). Future studies should aim to identify the mechanotransducers responsible for the relationship between flow and ROS regulation. Potential candidates include other GCX components (e.g., hyaluronic acid, chondroitin sulfate, sialic acid) or established mechanosensors such as Piezo1, G proteins, and caveolae, which have been previously implicated in endothelial activation [61, 62]. Additionally, further investigation into the intracellular signaling pathways downstream of HS is warranted to better understand how it modulates the endothelial inflammatory responses. Such work may also reveal how HS contributes to broader aspects of endothelial function, including permeability regulation and cell-to-cell communication, which are governed in part by the GCX [63, 64]. Combined with data presented in this paper, these future studies will help solidify the role of mechanotransduction in endothelial phenotype regulation while also highlighting the beneficial role of HS signaling in endothelial function.

## Supplementary Information

Below is the link to the electronic supplementary material.

Supplementary file1 (PDF 101 KB)

Supplementary file2 (PDF 609 KB)

## Data Availability

The data underlying this report is available within the main text, included in the supplemental figures document, and publicly accessible via the Dryad repository (10.5061/dryad.63xsj3vfg).

## Abbreviations

HS: Heparan sulfate
GCX: Glycocalyx
Hep III: Heparinase III
MFI: Mean fluorescence intensity
RNA-seq: RNA sequencing

## References

[1] Mudau, M., A. Genis, A. Lochner, and H. Strijdom. Endothelial dysfunction: the early predictor of atherosclerosis. *Cardiovasc J Afr*. 23(4):222–231, 2012. 10.5830/CVJA-2011-068. 22614668 10.5830/CVJA-2011-068 PMC3721957

[2] Sima, A. V., C. S. Stancu, and M. Simionescu. Vascular endothelium in atherosclerosis. *Cell Tissue Res*. 335(1):191–203, 2009. 10.1007/s00441-008-0678-5. 18797930 10.1007/s00441-008-0678-5

[3] Malyszko, J. Mechanism of endothelial dysfunction in chronic kidney disease. *Clin Chim Acta*. 411(19–20):1412–1420, 2010. 10.1016/j.cca.2010.06.019. 20598675 10.1016/j.cca.2010.06.019

[4] Rabelink, T. J., H. C. de Boer, and A. J. van Zonneveld. Endothelial activation and circulating markers of endothelial activation in kidney disease. *Nat Rev Nephrol*. 6(7):404–414, 2010. 10.1038/nrneph.2010.65. 20498676 10.1038/nrneph.2010.65

[5] Fassbender, K., T. Bertsch, O. Mielke, F. Muhlhauser, and M. Hennerici. Adhesion molecules in cerebrovascular diseases. Evidence for an inflammatory endothelial activation in cerebral large- and small-vessel disease. *Stroke*. 30(8):1647–1650, 1999. 10.1161/01.str.30.8.1647. 10436116 10.1161/01.str.30.8.1647

[6] Rouhl, R. P., J. G. Damoiseaux, J. Lodder, R. O. Theunissen, I. L. Knottnerus, J. Staals, L. H. Henskens, A. A. Kroon, P. W. de Leeuw, J. W. Tervaert, and R. J. van Oostenbrugge. Vascular inflammation in cerebral small vessel disease. *Neurobiol Aging*. 33(8):1800–1806, 2012. 10.1016/j.neurobiolaging.2011.04.008. 21601314 10.1016/j.neurobiolaging.2011.04.008

[7] Davies, M. J., J. L. Gordon, A. J. Gearing, R. Pigott, N. Woolf, D. Katz, and A. Kyriakopoulos. The expression of the adhesion molecules ICAM-1, VCAM-1, PECAM, and E-selectin in human atherosclerosis. *J Pathol*. 171(3):223–229, 1993. 10.1002/path.1711710311. 7506307 10.1002/path.1711710311

[8] Yang, L., R. M. Froio, T. E. Sciuto, A. M. Dvorak, R. Alon, and F. W. Luscinskas. ICAM-1 regulates neutrophil adhesion and transcellular migration of TNF-alpha-activated vascular endothelium under flow. *Blood*. 106(2):584–592, 2005. 10.1182/blood-2004-12-4942. 15811956 10.1182/blood-2004-12-4942 PMC1635241

[9] Hajra, L., A. I. Evans, M. Chen, S. J. Hyduk, T. Collins, and M. I. Cybulsky. The NF-kappa B signal transduction pathway in aortic endothelial cells is primed for activation in regions predisposed to atherosclerotic lesion formation. *Proc Natl Acad Sci U S A*. 97(16):9052–9057, 2000. 10.1073/pnas.97.16.9052. 10922059 10.1073/pnas.97.16.9052 PMC16820

[10] Read, M. A., M. Z. Whitley, A. J. Williams, and T. Collins. NF-kappa B and I kappa B alpha: an inducible regulatory system in endothelial activation. *J Exp Med*. 179(2):503–512, 1994. 10.1084/jem.179.2.503. 7507507 10.1084/jem.179.2.503 PMC2191350

[11] Frostegard, J., A. K. Ulfgren, P. Nyberg, U. Hedin, J. Swedenborg, U. Andersson, and G. K. Hansson. Cytokine expression in advanced human atherosclerotic plaques: dominance of pro-inflammatory (Th1) and macrophage-stimulating cytokines. *Atherosclerosis*. 145(1):33–43, 1999. 10.1016/s0021-9150(99)00011-8. 10428293 10.1016/s0021-9150(99)00011-8

[12] Fries, J. W., A. J. Williams, R. C. Atkins, W. Newman, M. F. Lipscomb, and T. Collins. Expression of VCAM-1 and E-selectin in an in vivo model of endothelial activation. *Am J Pathol*. 143(3):725–737, 1993. 7689792 PMC1887207

[13] Moyer, C. F., D. Sajuthi, H. Tulli, and J. K. Williams. Synthesis of IL-1 alpha and IL-1 beta by arterial cells in atherosclerosis. *Am J Pathol*. 138(4):951–960, 1991. 2012178 PMC1886091

[14] Guzik, T. J., N. E. West, E. Black, D. McDonald, C. Ratnatunga, R. Pillai, and K. M. Channon. Vascular superoxide production by NAD(P)H oxidase: association with endothelial dysfunction and clinical risk factors. *Circ Res*. 86(9):E85-90, 2000. 10.1161/01.res.86.9.e85. 10807876 10.1161/01.res.86.9.e85

[15] Witting, P. K., B. S. Rayner, B. J. Wu, N. A. Ellis, and R. Stocker. Hydrogen peroxide promotes endothelial dysfunction by stimulating multiple sources of superoxide anion radical production and decreasing nitric oxide bioavailability. *Cell Physiol Biochem*. 20(5):255–268, 2007. 10.1159/000107512. 17762155 10.1159/000107512

[16] Craige, S. M., S. Kant, and J. F. Keaney Jr. Reactive oxygen species in endothelial function - from disease to adaptation. *Circ J*. 79(6):1145–1155, 2015. 10.1253/circj.CJ-15-0464. 25986771 10.1253/circj.CJ-15-0464

[17] Mann, G. E., J. Niehueser-Saran, A. Watson, L. Gao, T. Ishii, P. de Winter, and R. C. Siow. Nrf2/ARE regulated antioxidant gene expression in endothelial and smooth muscle cells in oxidative stress: implications for atherosclerosis and preeclampsia. *Sheng Li Xue Bao*. 59(2):117–127, 2007. 17437032

[18] Chen, B., Y. Lu, Y. Chen, and J. Cheng. The role of Nrf2 in oxidative stress-induced endothelial injuries. *J Endocrinol*. 225(3):R83-99, 2015. 10.1530/JOE-14-0662. 25918130 10.1530/JOE-14-0662

[19] Kovac, S., P. R. Angelova, K. M. Holmstrom, Y. Zhang, A. T. Dinkova-Kostova, and A. Y. Abramov. Nrf2 regulates ROS production by mitochondria and NADPH oxidase. *Biochim Biophys Acta*. 1850(4):794–801, 2015. 10.1016/j.bbagen.2014.11.021. 25484314 10.1016/j.bbagen.2014.11.021 PMC4471129

[20] Fisslthaler, B., S. Dimmeler, C. Hermann, R. Busse, and I. Fleming. Phosphorylation and activation of the endothelial nitric oxide synthase by fluid shear stress. *Acta Physiol Scand*. 168(1):81–88, 2000. 10.1046/j.1365-201x.2000.00627.x. 10691783 10.1046/j.1365-201x.2000.00627.x

[21] Morigi, M., C. Zoja, M. Figliuzzi, M. Foppolo, G. Micheletti, M. Bontempelli, M. Saronni, G. Remuzzi, and A. Remuzzi. Fluid shear stress modulates surface expression of adhesion molecules by endothelial cells. *Blood*. 85(7):1696–1703, 1995. 7535583

[22] Hsieh, H. J., C. C. Cheng, S. T. Wu, J. J. Chiu, B. S. Wung, and D. L. Wang. Increase of reactive oxygen species (ROS) in endothelial cells by shear flow and involvement of ROS in shear-induced c-fos expression. *J Cell Physiol*. 175(2):156–162, 1998. 10.1002/(SICI)1097-4652(199805)175:2<156::AID-JCP5>3.0.CO;2-N. 9525474 10.1002/(SICI)1097-4652(199805)175:2<156::AID-JCP5>3.0.CO;2-N

[23] Breton-Romero, R., C. Gonzalez de Orduna, N. Romero, F. J. Sanchez-Gomez, C. de Alvaro, A. Porras, F. Rodriguez-Pascual, J. Laranjinha, R. Radi, and S. Lamas. Critical role of hydrogen peroxide signaling in the sequential activation of p38 MAPK and eNOS in laminar shear stress. *Free Radic Biol Med*. 52(6):1093–1100, 2012. 10.1016/j.freeradbiomed.2011.12.026. 22281399 10.1016/j.freeradbiomed.2011.12.026

[24] Han, Z., S. Varadharaj, R. J. Giedt, J. L. Zweier, H. H. Szeto, and B. R. Alevriadou. Mitochondria-derived reactive oxygen species mediate heme oxygenase-1 expression in sheared endothelial cells. *J Pharmacol Exp Ther*. 329(1):94–101, 2009. 10.1124/jpet.108.145557. 19131585 10.1124/jpet.108.145557 PMC2670602

[25] Subramanian, A., P. Tamayo, V. K. Mootha, S. Mukherjee, B. L. Ebert, M. A. Gillette, A. Paulovich, S. L. Pomeroy, T. R. Golub, E. S. Lander, and J. P. Mesirov. Gene set enrichment analysis: a knowledge-based approach for interpreting genome-wide expression profiles. *Proc Natl Acad Sci U S A*. 102(43):15545–15550, 2005. 10.1073/pnas.0506580102. 16199517 10.1073/pnas.0506580102 PMC1239896

[26] Wang, Y. X., H. B. Liu, P. S. Li, W. X. Yuan, B. Liu, S. T. Liu, and K. R. Qin. “ROS and NO Dynamics in Endothelial Cells Exposed to Exercise-Induced Wall Shear Stress,” (in eng). *Cell Mol Bioeng*. 12(1):107–120, 2019. 10.1007/s12195-018-00557-w. 31719902 10.1007/s12195-018-00557-w PMC6816775

[27] Hwang, J., A. Saha, Y. C. Boo, G. P. Sorescu, J. S. McNally, S. M. Holland, S. Dikalov, D. P. Giddens, K. K. Griendling, D. G. Harrison, and H. Jo. Oscillatory shear stress stimulates endothelial production of O2- from p47phox-dependent NAD(P)H oxidases, leading to monocyte adhesion. *J Biol Chem*. 278(47):47291–47298, 2003. 10.1074/jbc.M305150200. 12958309 10.1074/jbc.M305150200

[28] Mohan, S., K. Koyoma, A. Thangasamy, H. Nakano, R. D. Glickman, and N. Mohan. Low shear stress preferentially enhances IKK activity through selective sources of ROS for persistent activation of NF-kappaB in endothelial cells. *Am J Physiol Cell Physiol*. 292(1):C362–C371, 2007. 10.1152/ajpcell.00535.2005. 16914532 10.1152/ajpcell.00535.2005

[29] J. M. Tarbell and E. E. Ebong, "The Endothelial Glycocalyx: A Mechano-Sensor and -Transducer," (in English), *Science Signaling,* vol. 1, no. 40, Oct 7 2008.10.1126/scisignal.140pt8 10.1126/scisignal.140pt8 18840877

[30] Ebong, E. E., S. V. Lopez-Quintero, V. Rizzo, D. C. Spray, and J. M. Tarbell. “Shear-induced endothelial NOS activation and remodeling via heparan sulfate, glypican-1, and syndecan-1,” (in English). *Integrative Biology*. 6(3):338–347, 2014. 10.1039/c3ib40199e. 24480876 10.1039/c3ib40199e PMC3996848

[31] I. C. Harding, R. Mitra, S. A. Mensah, I. M. Herman, and E. E. Ebong, "Pro-atherosclerotic disturbed flow disrupts caveolin-1 expression, localization, and function via glycocalyx degradation," (in English), *Journal of Translational Medicine,* vol. 16, Dec 18 2018.10.1186/s12967-018-1721-2 10.1186/s12967-018-1721-2 PMC6299559 30563532

[32] McDonald, K. K., S. Cooper, L. Danielzak, and R. L. Leask. Glycocalyx Degradation Induces a Proinflammatory Phenotype and Increased Leukocyte Adhesion in Cultured Endothelial Cells under Flow. *PLoS One*.11(12):e0167576, 2016. 10.1371/journal.pone.0167576. 27907146 10.1371/journal.pone.0167576 PMC5132265

[33] Voyvodic, P. L., D. Min, R. Liu, E. Williams, V. Chitalia, A. K. Dunn, and A. B. Baker. “Loss of Syndecan-1 Induces a Pro-inflammatory Phenotype in Endothelial Cells with a Dysregulated Response to Atheroprotective Flow*,” (in English). *Journal of Biological Chemistry*. 289(14):9547–9559, 2014. 10.1074/jbc.M113.541573. 24554698 10.1074/jbc.M113.541573 PMC3975006

[34] Kumagai, R., X. Lu, and G. S. Kassab. Role of glycocalyx in flow-induced production of nitric oxide and reactive oxygen species. *Free Radic Biol Med*. 47(5):600–607, 2009. 10.1016/j.freeradbiomed.2009.05.034. 19500664 10.1016/j.freeradbiomed.2009.05.034 PMC2744202

[35] Fuster, M. M., and L. Wang. Endothelial heparan sulfate in angiogenesis. *Prog Mol Biol Transl Sci*. 93:179–212, 2010. 10.1016/S1877-1173(10)93009-3. 20807646 10.1016/S1877-1173(10)93009-3 PMC3703633

[36] Qiao, D., K. Meyer, C. Mundhenke, S. A. Drew, and A. Friedl. Heparan sulfate proteoglycans as regulators of fibroblast growth factor-2 signaling in brain endothelial cells. Specific role for glypican-1 in glioma angiogenesis. *J Biol Chem*. 278(18):16045–16053, 2003. 10.1074/jbc.M211259200. 12591930 10.1074/jbc.M211259200

[37] Ferreras, C., G. Rushton, C. L. Cole, M. Babur, B. A. Telfer, T. H. van Kuppevelt, J. M. Gardiner, K. J. Williams, G. C. Jayson, and E. Avizienyte. Endothelial heparan sulfate 6-O-sulfation levels regulate angiogenic responses of endothelial cells to fibroblast growth factor 2 and vascular endothelial growth factor. *J Biol Chem*. 287(43):36132–36146, 2012. 10.1074/jbc.M112.384875. 22927437 10.1074/jbc.M112.384875 PMC3476281

[38] Xu, D., J. Young, D. Song, and J. D. Esko. Heparan sulfate is essential for high mobility group protein 1 (HMGB1) signaling by the receptor for advanced glycation end products (RAGE). *J Biol Chem*. 286(48):41736–41744, 2011. 10.1074/jbc.M111.299685. 21990362 10.1074/jbc.M111.299685 PMC3308882

[39] Bao, X., E. A. Moseman, H. Saito, B. Petryniak, A. Thiriot, S. Hatakeyama, Y. Ito, H. Kawashima, Y. Yamaguchi, J. B. Lowe, U. H. von Andrian, and M. Fukuda. Endothelial heparan sulfate controls chemokine presentation in recruitment of lymphocytes and dendritic cells to lymph nodes. *Immunity*. 33(5):817–829, 2010. 10.1016/j.immuni.2010.10.018. 21093315 10.1016/j.immuni.2010.10.018 PMC2996097

[40] Mensah, S. A., A. A. Nersesyan, I. C. Harding, C. I. Lee, X. Tan, S. Banerjee, M. Niedre, V. P. Torchilin, and E. E. Ebong. Flow-regulated endothelial glycocalyx determines metastatic cancer cell activity. *FASEB J*. 34(5):6166–6184, 2020. 10.1096/fj.201901920R. 32167209 10.1096/fj.201901920R PMC7200301

[41] Peshavariya, H. M., G. J. Dusting, and S. Selemidis. Analysis of dihydroethidium fluorescence for the detection of intracellular and extracellular superoxide produced by NADPH oxidase. *Free Radic Res*. 41(6):699–712, 2007. 10.1080/10715760701297354. 17516243 10.1080/10715760701297354

[42] Oparka, M., J. Walczak, D. Malinska, L. van Oppen, J. Szczepanowska, W. J. H. Koopman, and M. R. Wieckowski. Quantifying ROS levels using CM-H2DCFDA and HyPer. *Methods*. 109:3–11, 2016. 10.1016/j.ymeth.2016.06.008. 27302663 10.1016/j.ymeth.2016.06.008

[43] Alom-Ruiz, S. P., N. Anilkumar, and A. M. Shah. Reactive oxygen species and endothelial activation. *Antioxid Redox Signal*. 10(6):1089–1100, 2008. 10.1089/ars.2007.2007. 18315494 10.1089/ars.2007.2007

[44] Ajami, N. E., S. Gupta, M. R. Maurya, P. Nguyen, J. Y. Li, J. Y. Shyy, Z. Chen, S. Chien, and S. Subramaniam. Systems biology analysis of longitudinal functional response of endothelial cells to shear stress. *Proc Natl Acad Sci U S A*. 114(41):10990–10995, 2017. 10.1073/pnas.1707517114. 28973892 10.1073/pnas.1707517114 PMC5642700

[45] Topper, J. N., and M. A. Gimbrone Jr. Blood flow and vascular gene expression: fluid shear stress as a modulator of endothelial phenotype. *Mol Med Today*. 5(1):40–46, 1999. 10.1016/s1357-4310(98)01372-0. 10088131 10.1016/s1357-4310(98)01372-0

[46] M. R. Maurya, S. Gupta, J. Y. Li, N. E. Ajami, Z. B. Chen, J. Y. Shyy, S. Chien, and S. Subramaniam, "Longitudinal shear stress response in human endothelial cells to atheroprone and atheroprotective conditions," (in eng), *Proc Natl Acad Sci U S A,* vol. 118, no. 4, Jan 26 2021.10.1073/pnas.2023236118 10.1073/pnas.2023236118 PMC7848718 33468662

[47] Q. Meng, L. Pu, M. Qi, S. Li, B. Sun, Y. Wang, B. Liu, and F. Li, "Laminar shear stress inhibits inflammation by activating autophagy in human aortic endothelial cells through HMGB1 nuclear translocation," (in eng), *Commun Biol,* vol. 5, no. 1, p. 425, May 6 2022.10.1038/s42003-022-03392-y 10.1038/s42003-022-03392-y PMC9076621 35523945

[48] R. P. Richter, A. R. Ashtekar, L. Zheng, D. Pretorius, T. Kaushlendra, R. D. Sanderson, A. Gaggar, and J. R. Richter, "Glycocalyx heparan sulfate cleavage promotes endothelial cell angiopoietin-2 expression by impairing shear stress-related AMPK/FoxO1 signaling," (in eng), *JCI Insight,* vol. 7, no. 15, Aug 8 2022.10.1172/jci.insight.155010 10.1172/jci.insight.155010 PMC9462499 35763350

[49] Richter, R. P., J. D. Odum, C. Margaroli, J. C. Cardenas, L. Zheng, K. Tripathi, Z. Wang, K. Arnold, R. D. Sanderson, J. Liu, and J. R. Richter. “Trauma promotes heparan sulfate modifications and cleavage that disrupt homeostatic gene expression in microvascular endothelial cells,” (in eng). *Front Cell Dev Biol*. 12:1390794, 2024. 10.3389/fcell.2024.1390794. 39114570 10.3389/fcell.2024.1390794 PMC11303185

[50] Montezano, A. C., and R. M. Touyz. Reactive oxygen species and endothelial function–role of nitric oxide synthase uncoupling and Nox family nicotinamide adenine dinucleotide phosphate oxidases. *Basic Clin Pharmacol Toxicol*. 110(1):87–94, 2012. 10.1111/j.1742-7843.2011.00785.x. 21883939 10.1111/j.1742-7843.2011.00785.x

[51] Niu, T., R. Xuan, L. Jiang, W. Wu, Z. Zhen, Y. Song, L. Hong, K. Zheng, J. Zhang, Q. Xu, Y. Tan, X. Yan, and H. Chen. Astaxanthin Induces the Nrf2/HO-1 Antioxidant Pathway in Human Umbilical Vein Endothelial Cells by Generating Trace Amounts of ROS. *J Agric Food Chem*. 66(6):1551–1559, 2018. 10.1021/acs.jafc.7b05493. 29381356 10.1021/acs.jafc.7b05493

[52] Lin, X., H. Yang, L. Zhou, and Z. Guo. Nrf2-dependent induction of NQO1 in mouse aortic endothelial cells overexpressing catalase. *Free Radic Biol Med*. 51(1):97–106, 2011. 10.1016/j.freeradbiomed.2011.04.020. 21569840 10.1016/j.freeradbiomed.2011.04.020 PMC3109219

[53] Warabi, E., W. Takabe, T. Minami, K. Inoue, K. Itoh, M. Yamamoto, T. Ishii, T. Kodama, and N. Noguchi. Shear stress stabilizes NF-E2-related factor 2 and induces antioxidant genes in endothelial cells: role of reactive oxygen/nitrogen species. *Free Radic Biol Med*. 42(2):260–269, 2007. 10.1016/j.freeradbiomed.2006.10.043. 17189831 10.1016/j.freeradbiomed.2006.10.043

[54] Jones, C. I., 3rd., H. Zhu, S. F. Martin, Z. Han, Y. Li, and B. R. Alevriadou. Regulation of antioxidants and phase 2 enzymes by shear-induced reactive oxygen species in endothelial cells. *Ann Biomed Eng*. 35(5):683–693, 2007. 10.1007/s10439-007-9279-9. 17340195 10.1007/s10439-007-9279-9

[55] L. Milkovic, N. Zarkovic, Z. Marusic, K. Zarkovic, and M. Jaganjac, "The 4-Hydroxynonenal-Protein Adducts and Their Biological Relevance: Are Some Proteins Preferred Targets?," (in eng), *Antioxidants (Basel),* vol. 12, no. 4, Apr 1 2023.10.3390/antiox12040856 10.3390/antiox12040856 PMC10135105 37107229

[56] Hayes, J. D., and R. C. Strange. “Glutathione S-transferase polymorphisms and their biological consequences,” (in eng). *Pharmacology*. 61(3):154–166, 2000. 10.1159/000028396. 10971201 10.1159/000028396

[57] Imai, H., M. Matsuoka, T. Kumagai, T. Sakamoto, and T. Koumura. “Lipid Peroxidation-Dependent Cell Death Regulated by GPx4 and Ferroptosis,” (in eng). *Curr Top Microbiol Immunol*. 403:143–170, 2017. 10.1007/82_2016_508. 28204974 10.1007/82_2016_508

[58] Perkins, A., K. J. Nelson, D. Parsonage, L. B. Poole, and P. A. Karplus. “Peroxiredoxins: guardians against oxidative stress and modulators of peroxide signaling,” (in eng). *Trends Biochem Sci*. 40(8):435–445, 2015. 10.1016/j.tibs.2015.05.001. 26067716 10.1016/j.tibs.2015.05.001 PMC4509974

[59] Lu, J., and A. Holmgren. “The thioredoxin antioxidant system,” (in eng). *Free Radic Biol Med*. 66:75–87, 2014. 10.1016/j.freeradbiomed.2013.07.036. 23899494 10.1016/j.freeradbiomed.2013.07.036

[60] Ma, Q. “Role of nrf2 in oxidative stress and toxicity,” (in eng). *Annu Rev Pharmacol Toxicol*. 53:401–426, 2013. 10.1146/annurev-pharmtox-011112-140320. 23294312 10.1146/annurev-pharmtox-011112-140320 PMC4680839

[61] Albarran-Juarez, J., A. Iring, S. Wang, S. Joseph, M. Grimm, B. Strilic, N. Wettschureck, T. F. Althoff, and S. Offermanns. Piezo1 and Gq/G11 promote endothelial inflammation depending on flow pattern and integrin activation. *J Exp Med*. 215(10):2655–2672, 2018. 10.1084/jem.20180483. 30194266 10.1084/jem.20180483 PMC6170174

[62] Engel, D., L. Beckers, E. Wijnands, T. Seijkens, D. Lievens, M. Drechsler, N. Gerdes, O. Soehnlein, M. J. Daemen, R. V. Stan, E. A. Biessen, and E. Lutgens. Caveolin-1 deficiency decreases atherosclerosis by hampering leukocyte influx into the arterial wall and generating a regulatory T-cell response. *FASEB J*. 25(11):3838–3848, 2011. 10.1096/fj.11-183350. 21795505 10.1096/fj.11-183350 PMC3672907

[63] Mensah, S. A., M. J. Cheng, H. Homayoni, B. D. Plouffe, A. J. Coury, and E. E. Ebong. Regeneration of glycocalyx by heparan sulfate and sphingosine 1-phosphate restores inter-endothelial communication. *PLoS One*.12(10):e0186116, 2017. 10.1371/journal.pone.0186116. 29023478 10.1371/journal.pone.0186116 PMC5638341

[64] Singh, A., S. C. Satchell, C. R. Neal, E. A. McKenzie, J. E. Tooke, and P. W. Mathieson. Glomerular endothelial glycocalyx constitutes a barrier to protein permeability. *J Am Soc Nephrol*. 18(11):2885–2893, 2007. 10.1681/ASN.2007010119. 17942961 10.1681/ASN.2007010119

