# Supplementary material for: Multimodal Profiling Reveals Distinct Endothelial Activation Pathways Regulated by Flow and Heparan Sulfate
Source: Cell Mol Bioeng. 2026 Feb 1;19(1):89–110. doi: 10.1007/s12195-026-00884-3 (PMC13031597; doi:10.1007/s12195-026-00884-3)
Supplement: Supplementary file 1 — Supplementary file1 (PDF 101 KB) [file 12195_2026_884_MOESM1_ESM.pdf]

Supplemental Materials and Methods

Multimodal Profiling Reveals Distinct Endothelial Activation Pathways Regulated by Flow and Heparan Sulfate

Ian C. Harding[1,†], Nicholas R. O'Hare[2,†], Ira M. Herman[3,4,5], Eno E. Ebong[1,2,6]

[1]Department of Bioengineering, Northeastern University, Boston, MA, USA
[2]Department of Chemical Engineering, Northeastern University, Boston, MA, USA
[3]Department of Genetics, Molecular and Developmental Biology, Tufts University School of Medicine, Boston, MA, USA
[4]Center for Innovations in Wound Healing Research, Tufts University School of Medicine, Boston, MA, USA
[5]Tissue Health Plus, Inc., Fort Worth, TX USA
[6]Department of Neuroscience, Albert Einstein College of Medicine, New York, NY, USA
[†]These authors made equal contributions to this work.

More RNA Sequencing Protocol Details

FASTQ files were aligned to human genome build hg38 using STAR (version 2.6.0c)[1]. Ensembl-Gene-level counts for non-mitochondrial genes were generated using featureCounts (Subread package, version 1.6.2) and Ensembl annotation build 92 (uniquely aligned proper pairs, same strand). Separately, SAMtools (version 1.9) was used to count reads aligning in proper pairs at least once to either strand of the mitochondrial chromosome (chrM) or to the sense or antisense strands of Ensembl loci of gene biotype "rRNA" or of non-mitochondrial RepeatMasker loci of class "rRNA" (as defined in the RepeatMasker track retrieved from the UCSC Table Browser). FASTQ quality was assessed using FastQC (version 0.11.7), and alignment quality was assessed using RSeQC (version 3.0.0). Variance-stabilizing transformation was accomplished using the varianceStabilizingTransformation function in the DESeq2 R package (version 1.23.10)[2]. Principal Component Analysis was performed using the prcomp R function with variance stabilizing transformed expression values that were z-normalized (set to a mean of zero and a standard deviation of one) across all samples within each gene. Differential expression was assessed using the Wald test implemented in the DESeq2 R package. Correction for multiple hypothesis testing was accomplished using the Benjamini-Hochberg false discovery rate (FDR). All analyses were performed using the R environment for statistical computing (version 3.6.0). Gene Set Enrichment Analysis (GSEA) (version 2.2.1)[3] was used to identify biological terms, pathways and processes that are coordinately up- or down-regulated within each pairwise comparison. The Entrez Gene identifiers of all genes in the Ensembl Gene annotation were ranked by the Wald statistic computed for each effect in two-factor model and for each pairwise comparison. Each ranked list was then used to perform pre-ranked GSEA analyses (default parameters with random seed 1234) using the Entrez Gene versions of the Hallmark, Biocarta, KEGG, Reactome, PID, Gene Ontology (GO), and transcription factor and microRNA motif gene sets were obtained from the Molecular Signatures Database (MSigDB), version 7.1[4]. From the gene sets, information was extracted regarding genes most relevant to endothelial function (based on literature evidence supporting their expression and function) and based on their relative expression as determined by RNA-seq (i.e. number of counts). Ultimately, the oxidant, antioxidant, pro-inflammatory, and anti-inflammatory gene sets contained 3, 39, 58, and 22 genes, respectively.

1. Dobin A, Davis CA, Schlesinger F, et al. STAR: ultrafast universal RNA-seq aligner. *Bioinformatics.* 2013;29(1):15-21.
2. Love MI, Huber W, Anders S. Moderated estimation of fold change and dispersion for RNA-seq data with DESeq2. *Genome Biol.* 2014;15(12):550.
3. Subramanian A, Tamayo P, Mootha VK, et al. Gene set enrichment analysis: a knowledge-based approach for interpreting genome-wide expression profiles. *Proc Natl Acad Sci U S A.* 2005;102(43):15545-15550.
4. Subramanian A, Kuehn H, Gould J, Tamayo P, Mesirov JP. GSEA-P: a desktop application for Gene Set Enrichment Analysis. *Bioinformatics.* 2007;23(23):3251-3253.
