# Supplementary material for: Multimodal Profiling Reveals Distinct Endothelial Activation Pathways Regulated by Flow and Heparan Sulfate
Source: Cell Mol Bioeng. 2026 Feb 1;19(1):89–110. doi: 10.1007/s12195-026-00884-3 (PMC13031597; doi:10.1007/s12195-026-00884-3)
Supplement: Supplementary file 2 — Supplementary file2 (PDF 609 KB) [file 12195_2026_884_MOESM2_ESM.pdf]

Supplemental Figures

Multimodal Profiling Reveals Distinct Endothelial Activation Pathways Regulated by Flow and Heparan Sulfate

Ian C. Harding[1,†], Nicholas R. O'Hare[2,†],
Ira M. Herman[3,4,5], Eno E. Ebong[1,2,6]

[1]Department of Bioengineering, Northeastern University, Boston, MA, USA
[2]Department of Chemical Engineering, Northeastern University, Boston, MA, USA
[3]Department of Genetics, Molecular and Developmental Biology,
Tufts University School of Medicine, Boston, MA, USA
[4]Center for Innovations in Wound Healing Research,
Tufts University School of Medicine, Boston, MA, USA
[5]Tissue Health Plus, Inc., Fort Worth, TX USA
[6]Department of Neuroscience, Albert Einstein College of Medicine, New York, NY, USA
[†]These authors made equal contributions to this work.

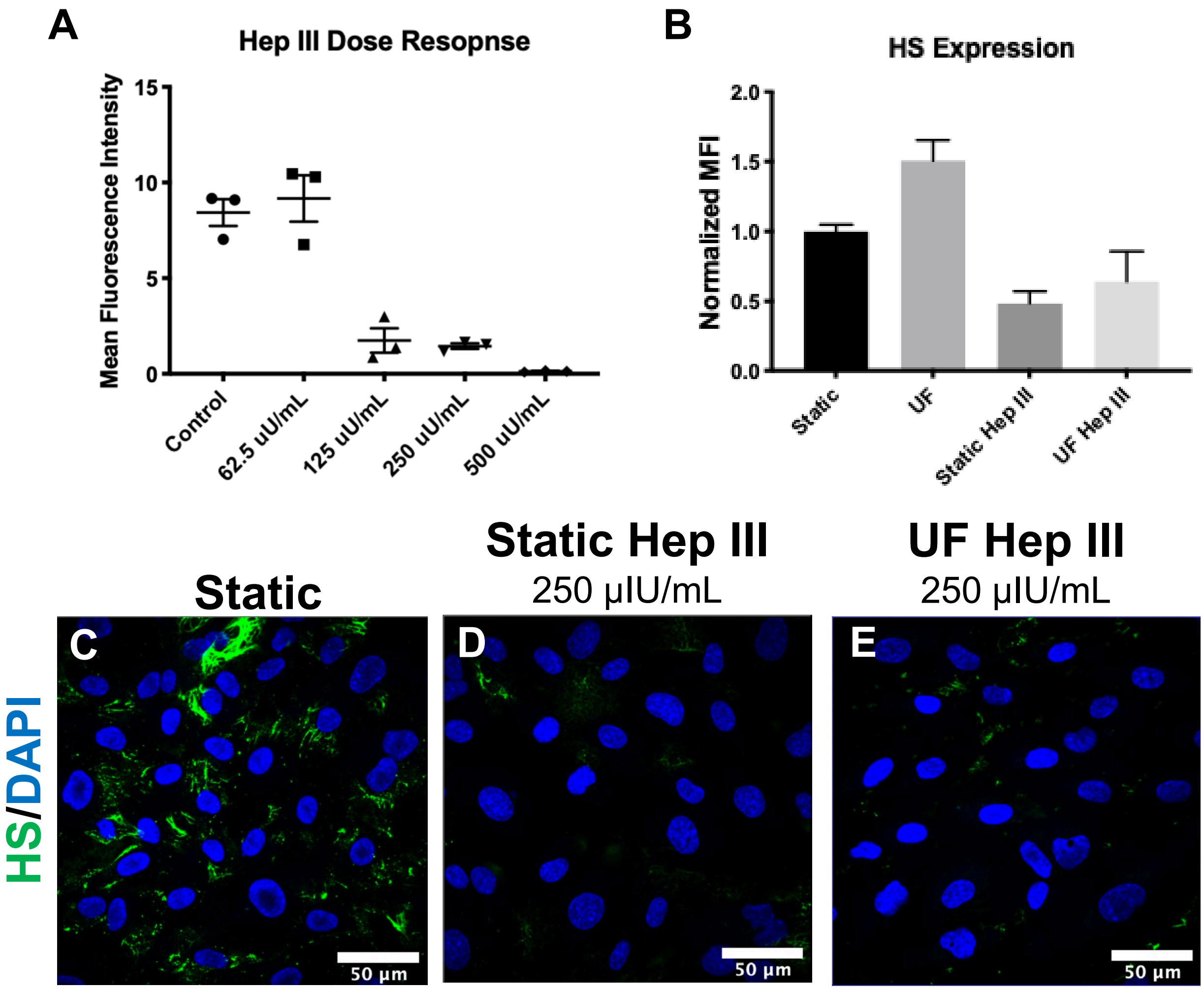

Supplemental Figure 1: *Confirmation of HS degradation after enzyme treatment.* (A) Quantification demonstrates a significant reduction in HS expression at increasing doses. Particularly, a substantial reduction is observed after 125 uIU/mL treatment. (B) A reduction in HS expression following Hep III treatment and after exposure to uniform flow was also confirmed. (C-E) HS immunocytochemistry demonstrates significant expression of HS in (C) control conditions, but not after Hep III treatment in either (D) static or (E) uniform flow conditions.

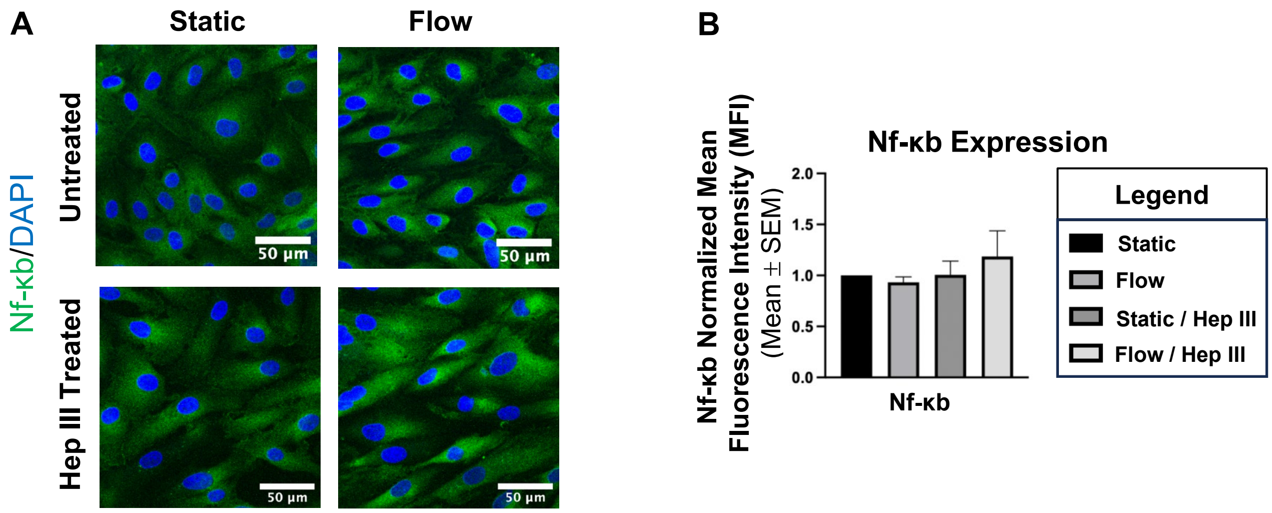

Supplemental Figure 2: *Nf-κb expression under static and flow conditions, in both the absence and presence of Hep III. Nf-κb was evaluated given its known role in bridging pro-inflammatory signaling and oxidative stress regulation.* (A) Fluorescent images of immunocytochemistry for the transcription factor Nf-κb show no changes in Nf-κb expression or activation (nuclear colocalization) after flow exposure or HS degradation. (B) Plot of MFI data confirms that Nf-κb fluorescence levels remain stable across static, flow, static/HepIII, and flow/HepIII conditions. Due to the absence of Nf-κb changes, the sample size was limited (n=2), and nuclear colocalization (an indicator of activation) was not quantified.

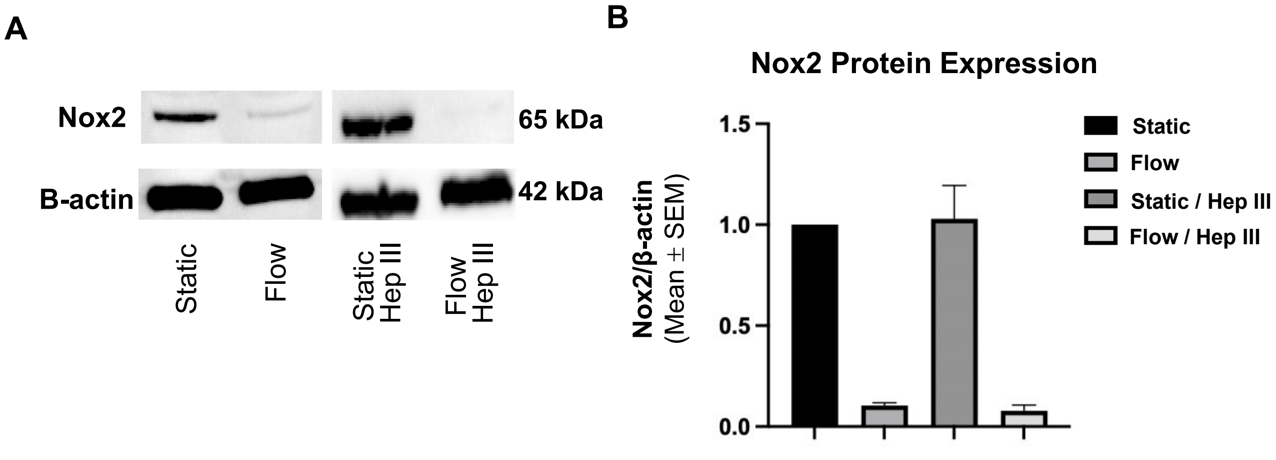

Supplemental Figure 3: *ROS precursor Nox2 data mirrors Nox4 data, with flow reducing its expression independent of HS expression.* (A) Western blots show a visible reduction in Nox2 expression following flow exposure, with no further change upon Hep III treatment. (B) Quantification of Nox2 quantification was based on static (n=3), flow (n=3), static/Hep III (n=2), and flow/Hep III (n=2) conditions. Nox2 data are included in supplemental figures for completeness but were excluded from main analyses due to limited replicates and insufficient statistical power. Experiments were discontinued early, as Nox4, its functional homolog, provided sufficient data to support study conclusions.

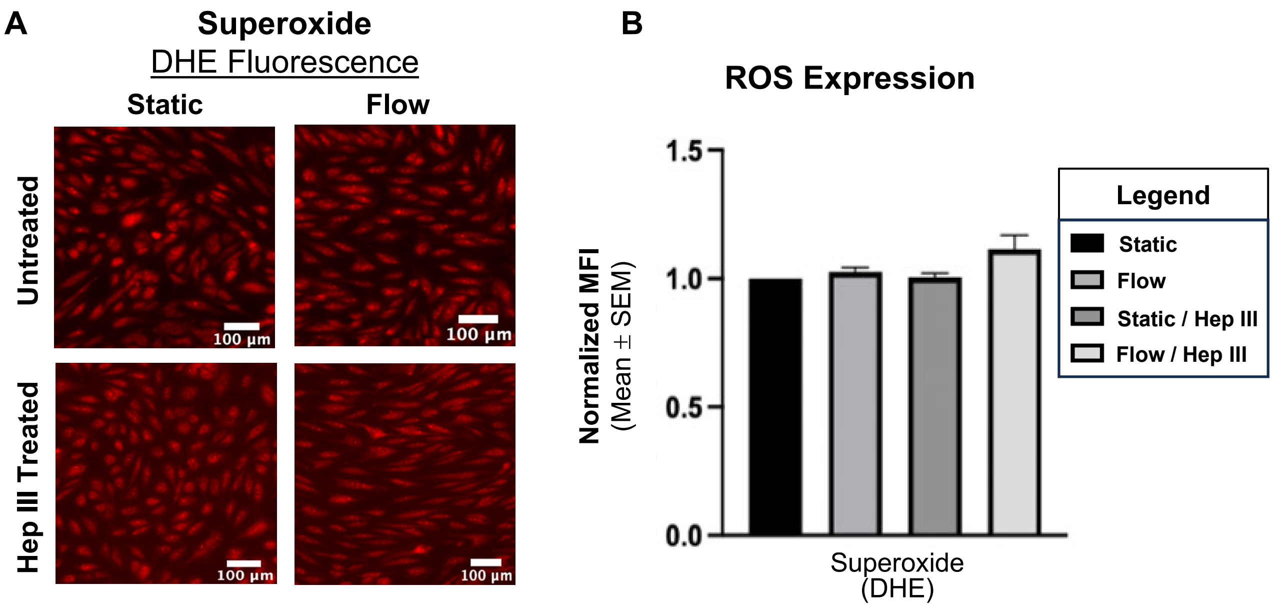

Supplemental Figure 4: *Fluorescent ROS assay performed using DHE, for the superoxide type of ROS.* (A) Representative fluorescence images showing results from the assay probing superoxide-type ROS using DHE. Neither flow nor HS degradation appear to affect superoxide levels. (B) MFI quantification from n = 6 samples. Statistical analyses confirmed no significant (ns) differences in superoxide levels across static, flow, static/HepIII, and flow/HepIII conditions.
